# Arginine starvation elicits chromatin leakage and cGAS-STING activation via epigenetic silencing of metabolic and DNA-repair genes

**DOI:** 10.7150/thno.54695

**Published:** 2021-06-04

**Authors:** Sheng-Chieh Hsu, Chia-Lin Chen, Mei-Ling Cheng, Cheng-Ying Chu, Chun A. Changou, Yen-Ling Yu, Shauh-Der Yeh, Tse-Chun Kuo, Cheng-Chin Kuo, Chih-Pin Chuu, Chien-Feng Li, Lu-Hai Wang, Hong-Wu Chen, Yun Yen, David K. Ann, Hung-Jung Wang, Hsing-Jien Kung

**Affiliations:** 1 Institute of Biotechnology, National Tsing Hua University, Hsinchu 30013, Taiwan; 2 Institute of Cellular and System Medicine, National Health Research Institutes, Miaoli County 35053, Taiwan; 3 Institute of Molecular and Genomic Medicine, National Health Research Institutes, Miaoli County 35053, Taiwan; 4 Metabolomics Core Laboratory, Healthy Aging Research Center, Chang Gung University, Taoyuan 33302, Taiwan; 5 Graduate Institute of Biomedical Sciences, College of Medicine, Chang Gung University, Taoyuan 33302, Taiwan; 6 Clinical Metabolomics Core Laboratory, Chang Gung Memorial Hospital at Linkou, Taoyuan 33305, Taiwan; 7 TMU Research Center of Cancer Translational Medicine, Taipei Medical University, Taipei 110, Taiwan; 8 CRISPR Gene Targeting Core Lab, Taipei Medical University, Taipei 110, Taiwan.; 9 The PhD Program for Translational Medicine, College of Medical Science and Technology, Taipei Medical University, Taipei 110, Taiwan; 10 The PhD Program for Cancer Molecular Biology and Drug Discovery, College of Medical Science and Technology, Taipei Medical University and Academia Sinica, Taipei 110, Taiwan; 11 Department of Urology, School of Medicine, College of Medicine, Taipei Medical University, Taipei 110, Taiwan; 12 Department of Urology, Taipei Medical University Hospital, Taipei 110, Taiwan; 13 Department of Pathology, Chi Mei Medical Center, Tainan City 73657, Taiwan; 14 Institute of Cancer Research, National Health Research Institutes, Miaoli County 35053, Taiwan; 15 Institute of Medical Science and Technology, National Sun Yat-sen University, Kaohsiung 80424, Taiwan; 16 Chinese Medicine Research Center and Institute of Integrated Medicine, China Medical University, Taichung 40402, Taiwan; 17 Department of Biochemistry and Molecular Medicine, School of Medicine, University of California, Davis, Sacramento, CA 95817, USA; 18 Department of Diabetes Complications and Metabolism, Beckman Research Institute, City of Hope, Duarte, CA 91010, USA; 19 Institute of Medical Sciences, Tzu Chi University, Hualien 97004, Taiwan; 20 Doctoral Degree Program in Translational Medicine, Tzu Chi University and Academia Sinica, Hualien 97004, Taiwan; 21 Ph.D. Program for Cancer Biology and Drug Discovery, College of Medical Science and Technology, Taipei Medical University, Taipei 110, Taiwan; 22 Department of Biochemistry and Molecular Medicine, Comprehensive Cancer Center, University of California, Davis, Sacramento, CA 95817, USA

**Keywords:** Arginine starvation, Epigenetic gene silencing, DNA leakage, cGAS-STING activation

## Abstract

**Rationale**: One of the most common metabolic defects in cancers is the deficiency in arginine synthesis, which has been exploited therapeutically. Yet, challenges remain, and the mechanisms of arginine-starvation induced killing are largely unclear. Here, we sought to demonstrate the underlying mechanisms by which arginine starvation-induced cell death and to develop a dietary arginine-restriction xenograft model to study the *in vivo* effects.

**Methods**: Multiple castration-resistant prostate cancer cell lines were treated with arginine starvation followed by comprehensive analysis of microarray, RNA-seq and ChIP-seq were to identify the molecular and epigenetic pathways affected by arginine starvation. Metabolomics and Seahorse Flux analyses were used to determine the metabolic profiles. A dietary arginine-restriction xenograft mouse model was developed to assess the effects of arginine starvation on tumor growth and inflammatory responses.

**Results**: We showed that arginine starvation coordinately and epigenetically suppressed gene expressions, including those involved in oxidative phosphorylation and DNA repair, resulting in DNA damage, chromatin-leakage and cGAS-STING activation, accompanied by the upregulation of type I interferon response. We further demonstrated that arginine starvation-caused depletion of α-ketoglutarate and inactivation of histone demethylases are the underlying causes of epigenetic silencing. Significantly, our dietary arginine-restriction model showed that arginine starvation suppressed prostate cancer growth *in vivo*, with evidence of enhanced interferon responses and recruitment of immune cells.

**Conclusions**: Arginine-starvation induces tumor cell killing by metabolite depletion and epigenetic silencing of metabolic genes, leading to DNA damage and chromatin leakage. The resulting cGAS-STING activation may further enhance these killing effects.

## Introduction

A critical step in tumor development and survival is metabolic adaptation to cope with rapid cell division, hypoxia, and a nutritionally deprived microenvironment 1. The Warburg effect which switches mitochondrial oxidative phosphorylation to aerobic glycolysis is only the tip of the iceberg, as different types of tumors have different metabolic requirements. Tumor-specific metabolic reprogramming, however, also exposes tumor cell vulnerabilities, which can be exploited therapeutically. Thus, many cancer cells, but not their normal counterparts, are “addicted” to external nutrients such as serine, leucine and arginine, and can thus be “starved” to death by targeted treatment 2-6. One distinguishing feature of starvation therapy is that its killing mechanisms are usually distinct from conventional therapies 2. As such, metabolic stress should effectively complement genomic stress-based therapies as an adjunctive agent.

Arginine is one of the three amino acids that can directly activate the mechanistic target of rapamycin (mTOR), which senses cell nutritional states 1 and has profound impact on protein, lipid, and nucleotide synthesis 7, 8. In addition, arginine is known to be a precursor to nitric oxide, polyamine and creatinine 9, 10. Yet arginine is a semi-essential amino acid that can be modulated without ill effects on the organism and has been used as a nutritional supplement or in depletion as a therapeutic intervention. The reason that arginine is semi-essential is due to normal cells' intrinsic ability to synthesize the molecule from citrulline via ASS1 (argininosuccinate synthase 1) and ASL (argininosuccinate lyase) 11. In a significant number of tumor cells of various cancer types, ASS1 expression is suppressed 12-14, creating cancer's most prevalent metabolic deficiency by rendering cancer cells “addicted” to external arginine and thus vulnerable to arginine-starvation therapy.

Arginine starvation by using arginine-metabolizing enzymes such as arginase, arginine decarboxylase and arginine deiminase (ADI) has received increasing attention as an intervention to treat ASS1-low cancers 15. ADI-PEG20 (pegylated ADI), in particular, has undergone at least 20 phase I/II clinical trials including prostate cancer, with excellent safety profiles 16. Recent trials in combination with other agents have shown medical benefits in a number of cancers 6, 17-19. Depending on cancer type and cell context, mechanisms associated with ADI-mediated cell killing vary: some rely on caspase-dependent apoptosis 6, 17, 20, while others involve caspase-independent apoptosis or autophagic death 21, 22.

In our work using prostate cancer as a model to study ADI-based therapeutic effects, we have found that castration resistant cell lines including CWR22Rv1 and PC3 are all sensitive to arginine starvation therapy, whereas immortalized prostate epithelial RWPE1 cell line which expresses a high level of ASS1 is completely resistant 21. ADI sensitivity is independent of androgen receptor status and applicable to castration and enzalutamide resistant cells as well. In addition, in these ASS1-low CWR22Rv1 prostate cancer cells, ADI induced a novel type of cell killing, which is caspase-independent, but autophagy-, mitochondria- and ROS-dependent 2, 21, 23. As such, arginine starvation synergizes with standard chemotherapies such as docetaxel that rely on caspase-dependent apoptosis pathways.

Our previous studies showed that ADI or arginine-starvation have profound effects on mitochondria, resulting in impaired OXPHOS functions and ROS generation which lead to nuclear DNA damage and membrane rupture 2, 5. At the same time, prolonged arginine starvation induces excessive autophagy, which captures the damaged chromatin in a phenomenon referred to as chromatin-autophagy or chromatophagy 2, 23. However, the detailed mechanisms and the pathways connecting these phenomena remain poorly understood.

In this study, we have characterized in detail the mechanism whereby arginine starvation impairs mitochondrial OXPHOS function and found that the coordinated silencing of the nuclear encoded OXPHOS genes via histone methylation is a major underlying cause. Impaired OXPHOS function generates excess ROS and damages nuclear DNA, resulting in chromatin leakage into the cytosol. The epigenetic silencing of DNA repair and synthesis genes exacerbates this process. We have also studied the kinetics and nature of leaked chromatins and reported that they induced cGAS-STING signaling and upregulated type I interferon response. Importantly, this leads to the recruitment of immune cells that potentially enhance the killing effect of a dietary arginine-restriction xenograft model. Together, these studies have uncovered arginine as a potent epigenetic regulator and suggested that arginine starvation affects both the tumor cells and tumor microenvironment.

## Results

### Arginine starvation induces chromatin DNA leakage

**Figure 1:**
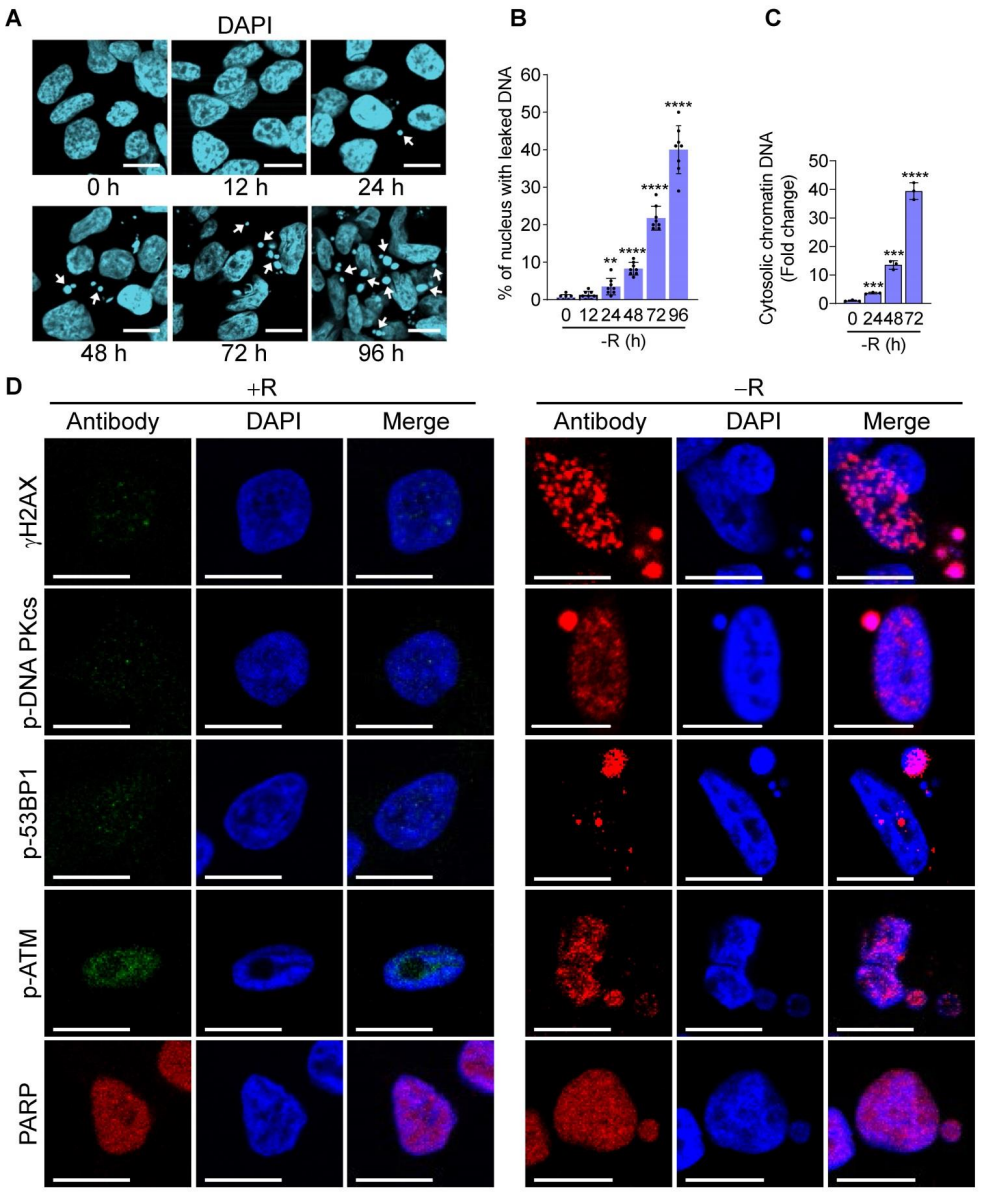
Arginine starvation induces severe chromatin leakage and DNA damage response. **(A)** CWR22Rv1 cells were cultured in arginine-free medium for indicated durations. Chromatin leakage was visualized with DAPI staining. Scale bars, 10 μm. **(B)** DAPI staining from random microscope fields was picked, and chromatin leakage was quantified by cell counting. (n = 8, **p < 0.01, ****p < 0.0001) **(C)** Cytosolic chromatin DNA was detected by the ELISA assay-based method as an indicator for chromatin leakage. (n = 3, ***p < 0.001, ****p < 0.0001) **(D)** DNA damage response was determined by immunostaining of γH2AX and various DNA repair machineries. Scale bars, 10 μm.

To fully appreciate the molecular pathways associated with arginine starvation-induced cell death, we characterized cellular changes of CWR22Rv1 and PC3 upon culturing in arginine-free media. Our previous work showed that ADI-PEG20 treatment of CRPC leads to leakage of nuclear DNA due to chromatophagy or chromatin-autophagy 2. We were able to recapitulate these phenomena in cells cultured in arginine-free medium with slightly faster kinetics. Nuclear DNA leakage started at around 24 h and increased afterwards (Figure 1A, arrowheads indicated). Using fluorescence microscopy to view randomly selected regions of interest (ROI), the ratio of nuclei with leaked DNAs to total nuclei consistently showed a time-dependent increase up to 96 h of arginine starvation (Figure 1B). With an ELISA-based assay to detect cytosolic chromatin-DNA (anti-DNAs and histone H3), we quantified cytosolic leaked DNA species and showed their increase in a time-dependent manner following arginine starvation in CWR22Rv1 (Figure 1C) and PC3 cells (Figure S1A).

Next, we wished to identify the nature of leaked nuclear DNA. We hypothesized that leaked nuclear DNA could be a consequence of genomic instability or deficiency of DNA repair activity under the arginine starvation condition, speculating that some of these are chromatins under repair. Confocal immunofluorescence microscopy analysis showed that γH2AX, a marker for DNA double-strand breaks appeared as punctate spots in the cell nucleus and enriched in leaked DNAs (Figure 1D). γH2AX upregulation was also presented in a time-dependent manner in both CWR22Rv1 and PC3 cells (Figures S1B-C), suggesting extensive DNA damage occurred in cells under arginine starvation. Consistently, we also found that several DNA repair-associated molecules, including p-DNA PKcs, p-53BP1, PARP, and p-ATM, were recruited to leaked DNAs (Figure 1D). These observations suggest that arginine starvation induces significant DNA damage and DNA repair responses leading to chromatin-leakage (indicated by the presence of histones). As will be shown below, this is likely caused by a combination of ROS-induced DNA damage with impaired DNA repair processes.

### Arginine starvation-induced leaked DNAs activate cGAS-STING pathway

**Figure 2:**
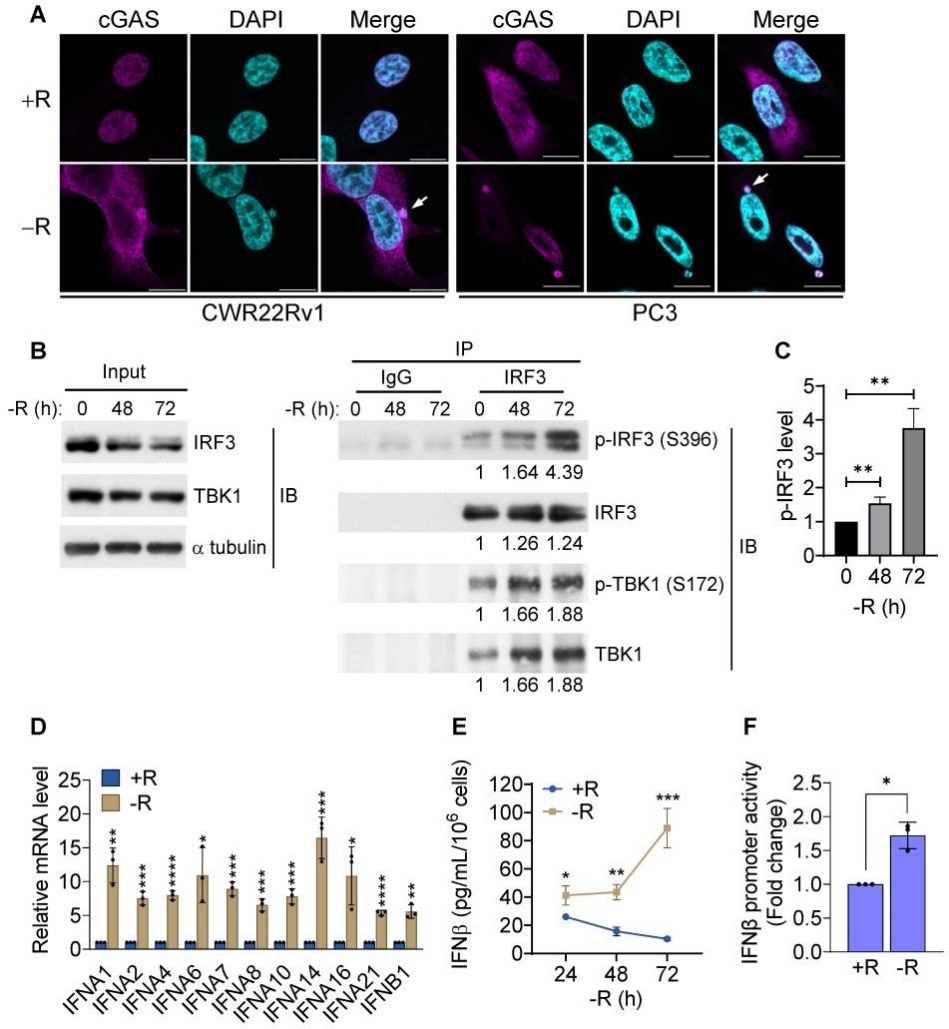
cGAS-STING pathway was activated by arginine starvation. **(A)** Cells overexpressing cGAS-V5 were deprived for 72 h and stained with anti-V5 antibodies. Scale bars, 10 μm. **(B)** CWR22Rv1 cells were deprived for indicate timepoints, and endogenous IRF3 was immunoprecipitated. IRF3 phosphorylation and TBK1 association were determined by immunoblots. Fold changes are listed below each blot. **(C)** IRF3 phosphorylation level was quantified and normalized with the IRF3. (n = 3, **p < 0.01). **(D)** RT-qPCR analysis of type I interferon expression in CWR22Rv1 cells starved for 72 h. (n = 3, *p < 0.05, **p < 0.01, ***p < 0.001, ****p < 0.0001) **(E)** IFNβ secretion by CWR22Rv1 cells after arginine starvation. (n = 3, *p < 0.05, **p < 0.01, ***p < 0.001) **(F)** IFNβ promoter activity in CWR22Rv1 cells deprived for 72 h. (n = 3, *p < 0.05)

It has been reported that genomic instability induced by irradiation or DNA repair inhibitor treatments could lead to nuclear DNA leakage with subsequent activation of DNA sensing receptors cGAS and its downstream STING (cGAS-STING) pathway 24. To determine whether arginine starvation-induced nuclear DNA leakage could lead to cGAS-STING activation, we examined whether cGAS could bind the leaked DNA by using confocal immunofluorescence microscopy. Indeed, cGAS was found to be colocalized with leaked DNA after 72 h of arginine starvation (Figure 2A), demonstrating that leaked DNA species induced by arginine starvation can trigger cGAS-dependent DNA sensing pathways.

**Figure 3:**
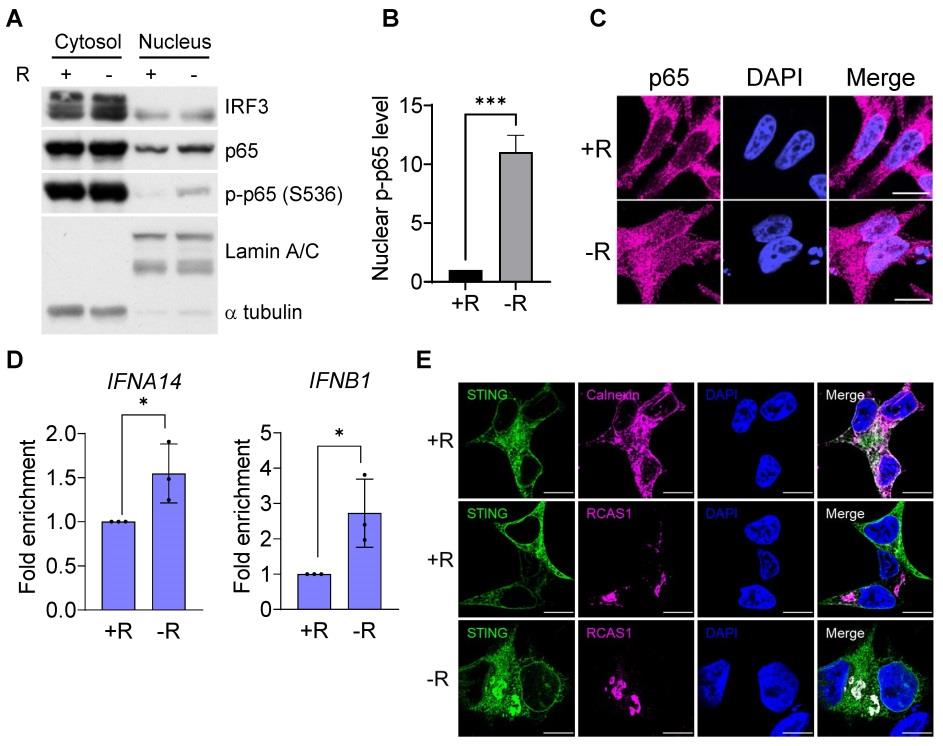
Translocation of the cGAS downstream effectors. **(A)** Nuclear translocation of p65 was determined by subcellular fractionation in arginine-deprived CWR22Rv1 cells. **(B)** Nuclear p65 was quantified and normalized to lamin A/C. (n = 3, ***p < 0.001) **(C)** Immunostaining of p65 in CWR22Rv1 cells. Scale bars, 10 μm. **(D)** ChIP-qPCR analysis of IRF3 binding on the promoter regions of *IFNA14* and *IFNB1* in CWR22Rv1. (n = 3, *p < 0.05) **(E)** CWR22Rv1 cells overexpressing STING-V5 were subjected to arginine deprivation and stained with V5, calnexin and RCAS1 antibodies. Scale bars, 10 μm.

To further investigate whether the DNA sensing cGAS-STING pathway was activated, we measured cGAS-STING signaling pathway hallmarks, including the cGAS-STING-dependent phosphorylation of TBK1 and IRF3, expression of type I interferon, and translocation of p65 NFκB and STING. Our data clearly showed that the phosphorylation levels of IRF3 and its associated TBK1 were increased after 72 h of arginine starvation in CWR22Rv1 cells (Figures 2B-C), with the consequent elevation of the transcription of type I interferon genes (Figure 2D). On the other hand, disrupting of cGAS-STING pathway by treatments of RU.521, a cGAS specific inhibitor, or cGAS-specific shRNA significantly attenuated the type I IFNs response (Figures S2A-B) and the level of IRF3 phosphorylation (Figure S2C). In addition, both the secreted IFNβ protein levels (Figure 2E) and luciferase-reporter activity of IFNβ (Figure 2F) were significantly increased in response to arginine starvation. Moreover, the nuclear translocation and levels of phosphorylation of p65 NFκB were increased under arginine starvation (Figures 3A-C). In the meantime, occupancy of IRF3 on the promoter regions of IFNA14 and IFNB1 was also increased (Figure 3D). STING translocation from the ER (marked by calnexin) to peri-nuclear trans-golgi (marked by RCAS1) regions 25 was also detected (Figure 3E). This data suggested arginine starvation-induced leaked DNA activates cGAS-STING signaling.

### Arginine starvation impairs mitochondrial OXPHOS functions and induces ROS

**Figure 4:**
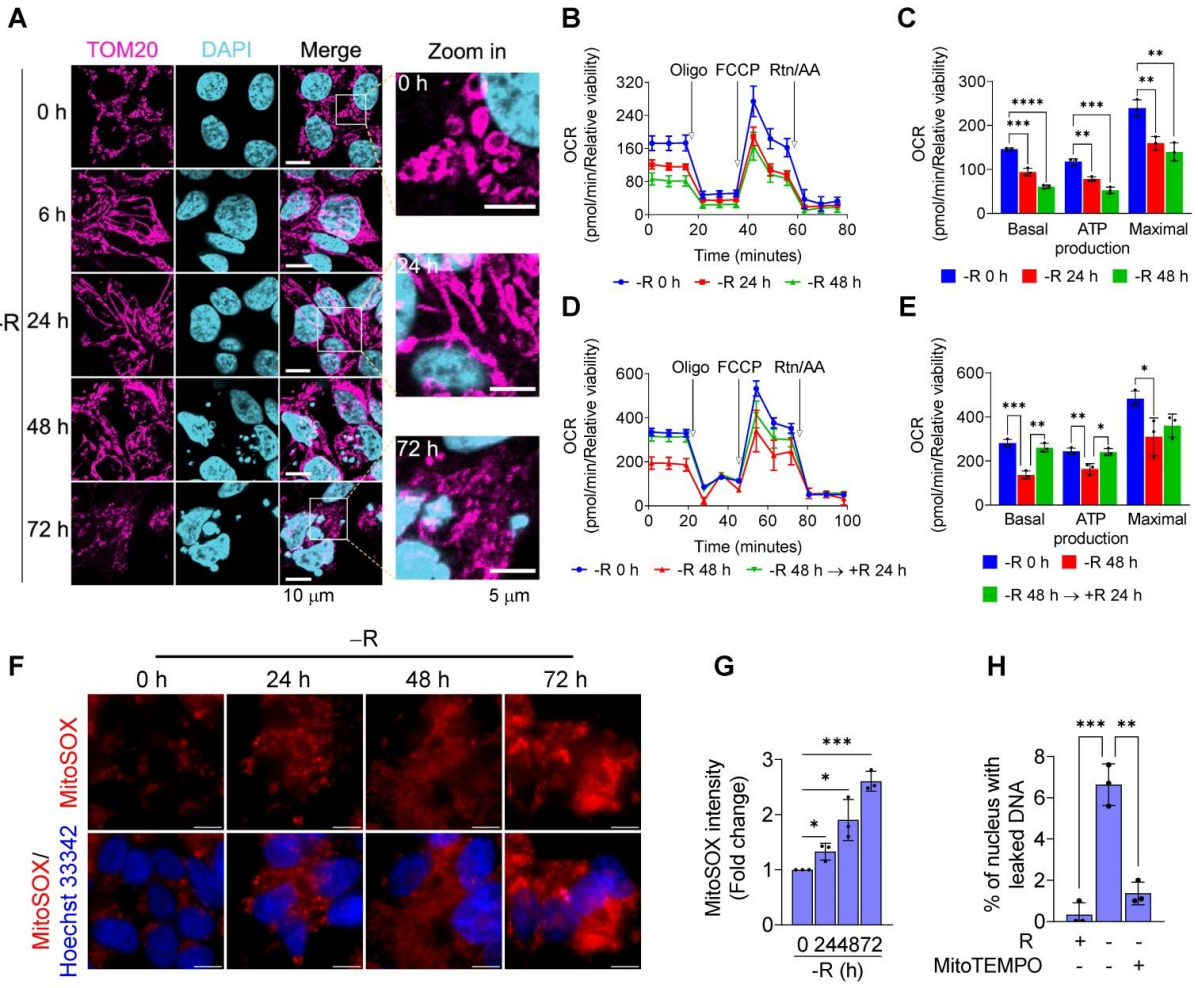
Arginine starvation altered mitochondrial dynamics and induced mitochondrial dysfunction. **(A)** Mitochondrial dynamics was visualized by immunostaining of the mitochondrial outer membrane protein, TOM20. **(B)** Oxygen consumption rate of arginine-deprived CWR22Rv1 cells. **(C)** Basal respiration, ATP production and maximal respiration in CWR22Rv1 after arginine starvation. (n = 3, **p < 0.01, ***p < 0.001, ****p < 0.0001). **(D)** CWR22Rv1 cells were deprived for 48 h, followed by reintroducing arginine for another 24 h. Oxygen consumption rate was determined by Seahorse assay. **(E)** Basal respiration, ATP production and maximal respiration in CWR22Rv1 after arginine reintroduction. (n = 3, *p < 0.05, **p < 0.01, ***p < 0.001) **(F)** Live images of mitochondrial ROS probed with MitoSOX. Scale bars, 10 μm. **(G)** Mitochondrial ROS was probed by MitoSOX, and fluorescent intensity was quantified. (n = 3, *p < 0.05, ***p < 0.001) **(H)** MitoTEMPO was employed to scavenge mitochondrial ROS, and chromatin leakage was quantified by cell counting. (n = 3, **p < 0.01, ***p < 0.001)

To look for a potential source of significant DNA damage causing chromatin leakage upon arginine starvation, we first characterized alterations of mitochondria, as its dysfunction and OXPHOS imbalance often lead to ROS generation 26. Immunofluorescence microscopy with antibodies against TOM20 (a translocase of outer membrane of mitochondria) at different time points of arginine starvation in CWR22Rv1 cell line revealed dynamic alterations of mitochondrial morphology (Figure 4A). At early time points (6 and 24 h) of treatment, elongated mitochondria (mostly the fusion phenotype) were observed. After a prolonged treatment (72 h), arginine starvation resulted in fragmented mitochondria (fission) and reduced OPA1 protein levels (Figure S3A) in parallel with nuclear DNA leakage.

We next measured the affected functions of mitochondria in response to arginine starvation by using Seahorse Bioscience Flux Analyzer. We found that oxygen consumption rates (OCRs) were reduced upon arginine starvation in a time-dependent manner (Figures 4B-C) and, furthermore, the attenuated OCRs, basal respiration and ATP production could be partially restored by adding back arginine (Figures 4D-E). These observations suggest that arginine starvation significantly interferes with cellular metabolic homeostasis and energy balance.

It has been known that impaired mitochondria are associated with over-production of mitochondrial ROS (mtROS) 26. To evaluate the levels of mtROS upon arginine starvation, we probed cellular mtROS with MitoSOX Red, a mitochondrial superoxide indicator, in combination with fluorescence microscopy, and revealed that arginine starvation could induce a time-dependent mtROS production (Figures 4F-G), findings in agreement with our measurement of mitochondrial dysfunction during arginine starvation. Same phenomenon was also observed in PC3 cells (Figure S3B). Importantly, removal of mtROS with mitochondrial-targeting ROS scavenger, MitoTEMPO, reduced the arginine starvation-induced DNA leakage (Figure 4H).

### Arginine starvation coordinately downmodulates mitochondrial and DNA repair-related gene expression

**Figure 5:**
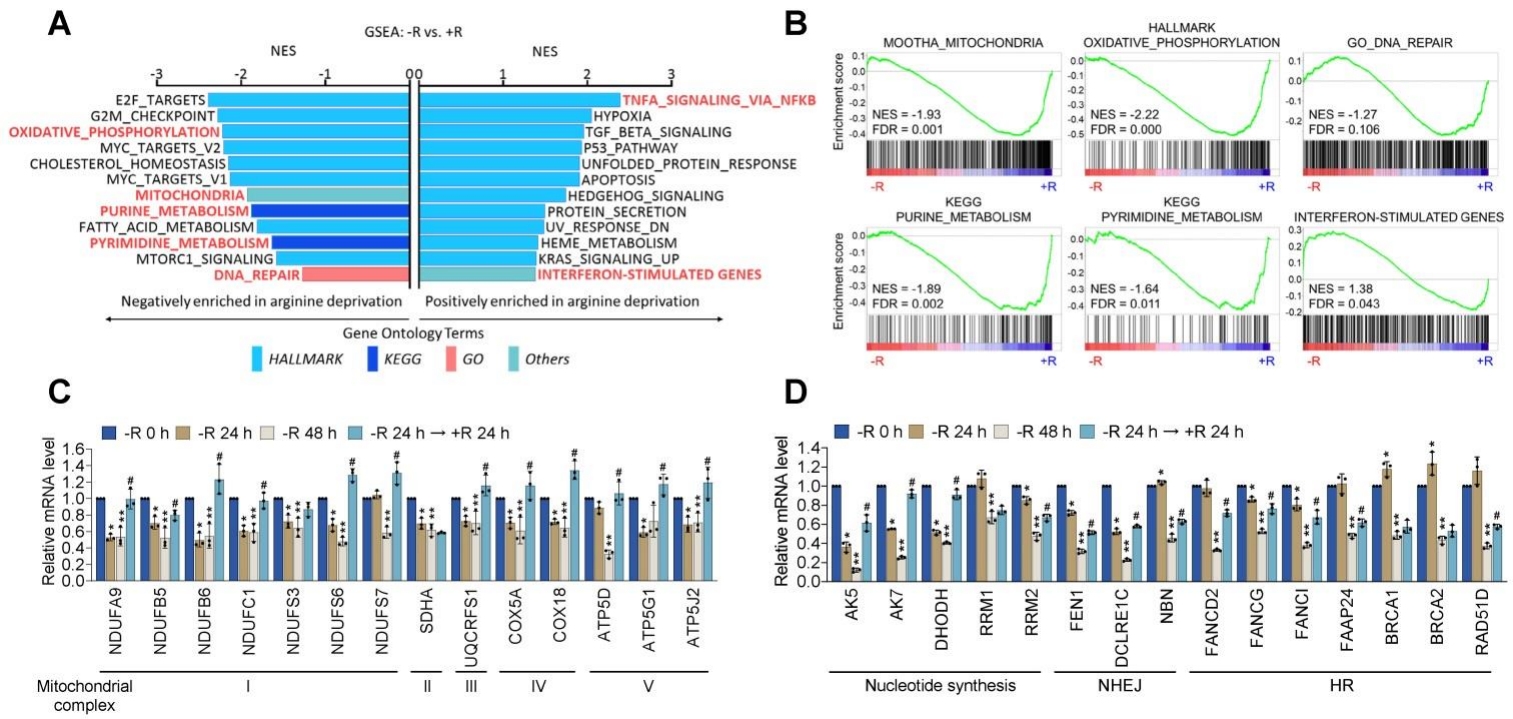
Arginine starvation downregulated OXPHOS and DNA-repair genes. **(A)** CWR22Rv1 cells were deprived of arginine for 48 h and subjected to GSEA analysis. All enrichment results met the statistical criteria of p < 0.05 and FDR < 25%. **(B)** Enrichment score plots of selected gene sets were presented. **(C)** OXPHOS gene expression. (* represents -R 24 h vs -R 0 h, ** represents -R 48 h vs -R 0 h, and # represents -R 24 h → +R 24 h vs -R 48 h. n = 3, *^,^ **^, #^p < 0.05) **(D)** Nucleotide metabolism and DNA-repair gene expression. (* represents -R 24 h vs -R 0 h, ** represents -R 48 h vs -R 0 h, and # represents -R 24 h → +R 24 h vs -R 48 h. n = 3, *^,^ **^, #^p < 0.05)

Deficiency in DNA repair machinery potentially leads to genome instability and nuclear DNA leakage 24. The production of mtROS coupled with the impaired DNA repair activity could account for the nuclear DNA leakage phenotypes upon arginine starvation. To explore the mechanisms responsible for arginine starvation-induced impaired mitochondrial functions and DNA repair activities, we performed microarray analysis of CWR22Rv1 cells treated with arginine starvation for 48 h (Figure S4A). GSEA (Gene Set Enrichment Analysis) results showed that metabolic pathways including OXPHOS, mitochondria, and the mTORC1 signaling pathways were significantly downmodulated (Figure 5A), consistent with decreased mitochondrial OCRs. We also found that DNA repair-related pathways including G2M checkpoint, purine and pyrimidine biosynthesis as well as mitotic spindle assembly pathways were downregulated by arginine starvation (Figure 5B), which may account for genome instability and a potential source of nuclear DNA leakage. We also confirmed the arginine-starvation downmodulated genes by RT-qPCR (Figures 5C-D). Importantly, the downmodulation pattern in the majority of these genes can be reversed by the addition of arginine, indicating a direct consequence of arginine starvation (Figure 5C-D). Similar gene expression profiles were also observed in PC3 cells (Figures S4B-C), indicating the finding is not unique to CWR22Rv1. Taken together, our results indicated that arginine starvation coordinately downregulated metabolic and DNA-repair gene expressions.

### Arginine starvation induces chromatin remodeling by increasing repressive histone methylations

**Figure 6:**
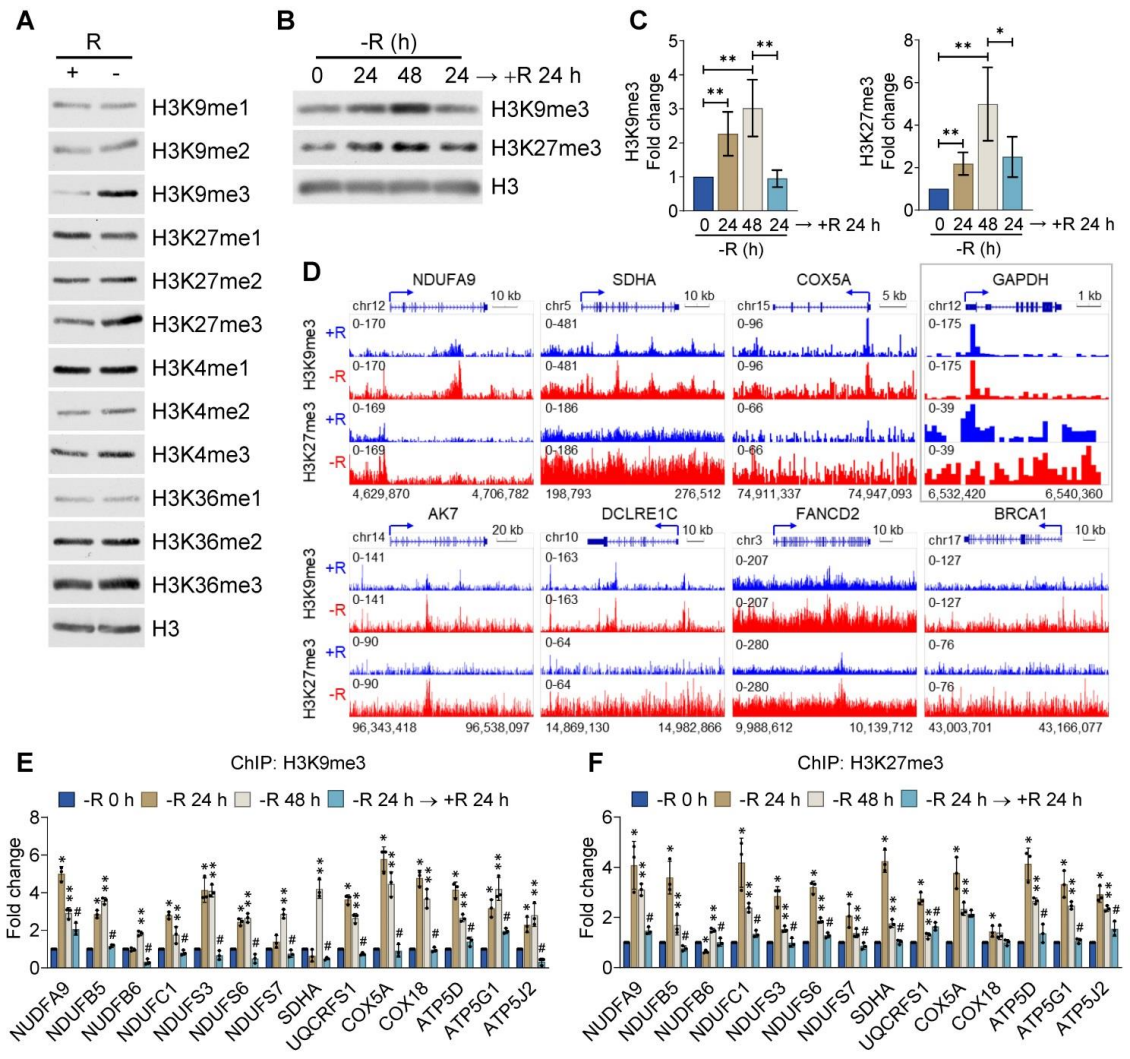
H3K9me3 and H3K27me3 were enhanced by arginine starvation. **(A)** CWR22Rv1 cells were deprived of arginine by ADI-PEG20. Histone lysine methylation status was analyzed by immunoblots. **(B)** CWR22Rv1 cells were maintained in arginine-free medium for indicated durations. H3K9me3 and H3K27me3 were analyzed with immunoblots. **(C)** Relative H3K9me3 and H3K27me3 levels were quantified and normalized to H3. (n = 3, *p < 0.05, **p < 0.01) **(D)** CWR22Rv1 cells were cultured in arginine-depleted medium and subjected to ChIP-seq analysis with anti-H3K9me3 and anti-H3K27me3 antibodies. **(E)** H3K9me3 occupancy on the promoter regions of OXPHOS genes was determined by ChIP-qPCR. (n = 3, * represents -R 24 h vs -R 0 h, ** represents -R 48 h vs -R 0 h, and # represents -R 24 h → +R 24 h vs -R 48 h. n = 3, *^,^ **^, #^p < 0.05) **(F)** H3K27me3 occupancy on the promoter regions of OXPHOS genes was determined by ChIP-qPCR. (n = 3, * represents -R 24 h vs -R 0 h, ** represents -R 48 h vs -R0h, and # represents -R 24 h → +R 24 h vs -R 48 h. n = 3, *^,^ **^, #^p < 0.05)

To reveal the underlying mechanism by which arginine starvation globally and coordinately downmodulated gene expression, we speculated that an epigenetic suppressive mechanism may operate in this process. To test this, we performed immunoblotting using specific antibodies against different histone modifications in CWR22Rv1 cells treated with arginine starvation for 48 h. We measured the levels of mono-, di- and tri-methylation of H3K4, H3K9, H3K27 and H3K36 respectively and found that arginine starvation significantly increased the levels of tri-methylation of histone H3K9 and H3K27 (Figure 6A), the major suppressive histone marks. The levels of mono- and di-methylation of H3K4, H3K9, H3K27 and H3K36 were also increased under arginine starvation, but to a lesser extent, suggesting an epigenetic regulatory mechanism that affected gene expression during arginine starvation. In agreement, our immunofluorescence analysis confirmed that H3K9me3 and H3K27me3 were significantly elevated compared with the controls (Figure S5A). Moreover, arginine starvation-induced upregulation of H3K9me3 and H3K27me3 could be observed in the treatment with ADI-PEG20 (Figure S5B). The evaluated levels of H3K9me3 and H3K27me3 can be restored by adding back arginine (Figures 6B-C) or direct removal of ADI-PEG20 (Figure S5B), indicating the specific effect of arginine starvation on the acquisition of repressive histone marks. This epigenetic suppression phenomenon was also observed in PC3 and MDA-MB-231 cells (Figures S5C-E).

To comprehensively characterize the effect of arginine starvation on gene expression, we performed chromatin immunoprecipitation-sequencing (ChIP-Seq) for H3K9me3 and H3K27me3 in CWR22Rv1 cells. The promoters of OXPHOS and DNA-repair genes, such as NDUFA9, SDHA, COX5A, DCLRE1C, FANCD2 and BRCA1 were significantly enriched with H3K9me3 and H3K27me3 upon arginine starvation (Figure 6D). By contrast, the promoter of GAPDH was less affected (Figure 6D, box). We further confirmed the increased recruitment of these repressive markers to the promoters of OXPHOS genes by ChIP-qPCR after 24 h or 48 h of arginine starvation, with histone H3 as a control (Figure 6E-F). In addition, arginine starvation-induced H3K9me3 enrichments in the DNA-repair gene promoters were also confirmed (Figure S5F). Importantly, increased enrichments of histone methylations can be restored by reintroducing arginine. Taken together, our results provide evidence that arginine starvation induces coordinated downmodulation of OXPHOS genes via epigenetic silencing mechanisms, mainly by increasing the level of both H3K9me3 and H3K27me3 on the target genes.

### Arginine starvation consumes intermediate metabolites of glycolysis and the TCA cycle

**Figure 7:**
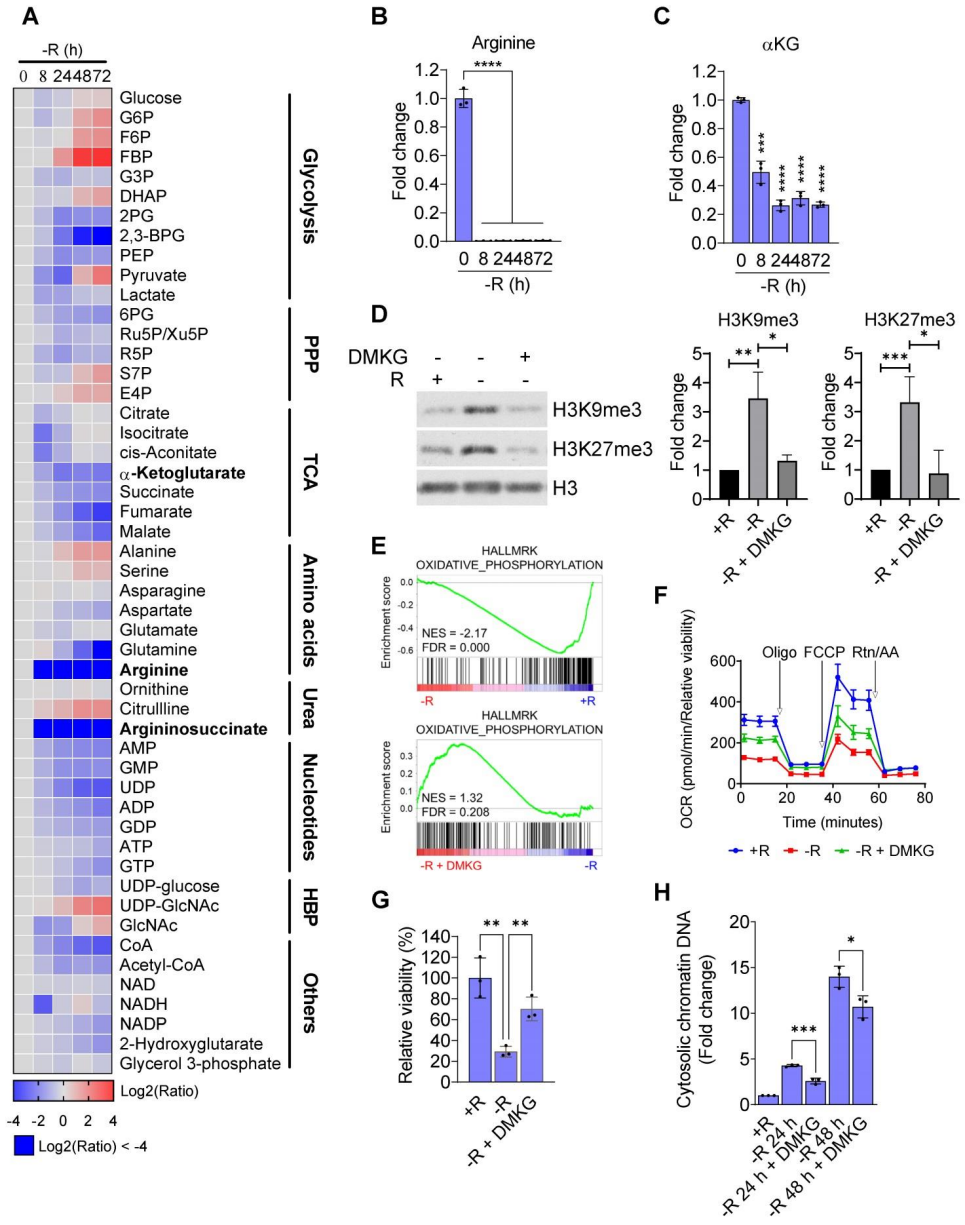
Arginine starvation reduced intracellular α-ketoglutarate. **(A)** Metabolites in arginine-deprived CWR22Rv1 cells were extracted and analyzed by UPLC/MS/MS. (n = 3) **(B)** Arginine levels were determined at each timepoint after arginine starvation. (n = 3, ****p < 0.0001) **(C)** Intracellular αKG level after arginine starvation in CWR22Rv1. (n = 3, ***p < 0.001, ****p < 0.0001) **(D)** H3K9me3 and H3K27me3 levels in arginine-deprived CWR22Rv1 with or without DMKG. The quantification results are shown in the right panel. (n = 3, *p < 0.05, **p < 0.01, ***p < 0.001) **(E)** GSEA analysis of arginine-deprived CWR22Rv1 with or without DMKG. **(F)** Oxygen consumption rate in arginine-deprived CWR22Rv1 with or without DMKG. **(G)** CWR22Rv1 cells were cultured in arginine-depleted medium with or without DMKG. Relative viability was determined with CCK-8 assay. (n = 3, **p < 0.01) **(H)** Cytosolic chromatin-DNA was quantified by ELISA assay. (n = 3, *p < 0.05, ***p < 0.001)

Since arginine starvation coordinately downregulated genes related to metabolic pathways, we speculated that cellular metabolites in the arginine-deprived cells could be involved. We therefore applied a metabolomics approach to profile cellular metabolites in the arginine-deprived CWR22Rv1 cells. We confirmed that both intracellular levels of arginine and argininosuccinate, metabolites of the urea cycle, declined dramatically as early as 8 h of starvation (Figure 7A-B). Furthermore, after 24 h of arginine starvation cellular metabolites of the TCA cycle, urea cycle, and glycolysis were also significantly decreased. We noted that the level of α-ketoglutarate (αKG), a critical cofactor for histone demethylation 27, was reduced in a time-dependent manner by arginine starvation (Figure 7C). Similarly, treatment of arginine starvation in PC3 cells also resulted in a decrease of αKG (Table S1).

As αKG is an obligate substrate for Jumonji-domain containing lysine demethylases (KDMs), depletion of αKG through arginine starvation could impair their activities leading to an elevation of histone methylations. To test this hypothesis, we reintroduced DMKG, a membrane permeable analog of αKG, into arginine-starvation condition. The addition of DMKG to the arginine-free media suppressed the arginine starvation-induced overall levels of H3K9me3 and H3K27me3 (Figure 7D). The ChIP-qPCR analysis also revealed that DMKG addition reversed the increased levels of both H3K9me3 and H3K27me3 on the promoters of OXPHOS genes (Figure S6A). GSEA analysis of the microarray data for DMKG-rescued CWR22Rv1 cells showed that OXPHOS genes downregulated by arginine starvation were upregulated (Figure 7E). The result was further confirmed by RT-qPCR (Figure S6B). OXPHOS activities were partially restored by DMKG using Seahorse bioenergetic flux assay (Figures 7F and S6C). Importantly, cell viability of the arginine-deprived CWR22Rv1 cells was significantly increased by 70% (Figure 7G), suggesting that αKG depletion plays a key role in cell death induced by arginine starvation. Finally, DMKG addition reduced the DNA damage marker γH2AX (Figure S6D) as well as the levels of nuclear DNA leakage induced by arginine-starvation (Figures 7H and S6E). Taken together, our results indicated that the αKG depletion causes epigenetic silencing of OXPHOS and DNA-repair genes, resulting in ROS-induced DNA damage and nuclear DNA leakage during arginine-starvation.

### Dietary restriction of arginine suppresses prostate cancer growth with inflammatory responses

We have previously shown that arginine metabolizing arginine deiminase (ADI-PEG20) is effective and tumor-selective in inhibiting growth of castration resistant prostate cancer (CRPC) xenografts 21 and displays synergism with docetaxel 21, 28. ADI-PEG20 induced autophagic and apoptotic cell death in ASS1-low prostate cancer cell lines including CWR22Rv1 and PC3. In the present study, instead of using ADI therapeutic starvation, we wished to test whether dietary restriction with an arginine-free diet would also act as an effective intervention, while also allowing study of the effects of external arginine (of lack thereof) directly on tumor growth, without the possible complication by metabolic products such as citrulline and ammonia 29.

**Figure 8:**
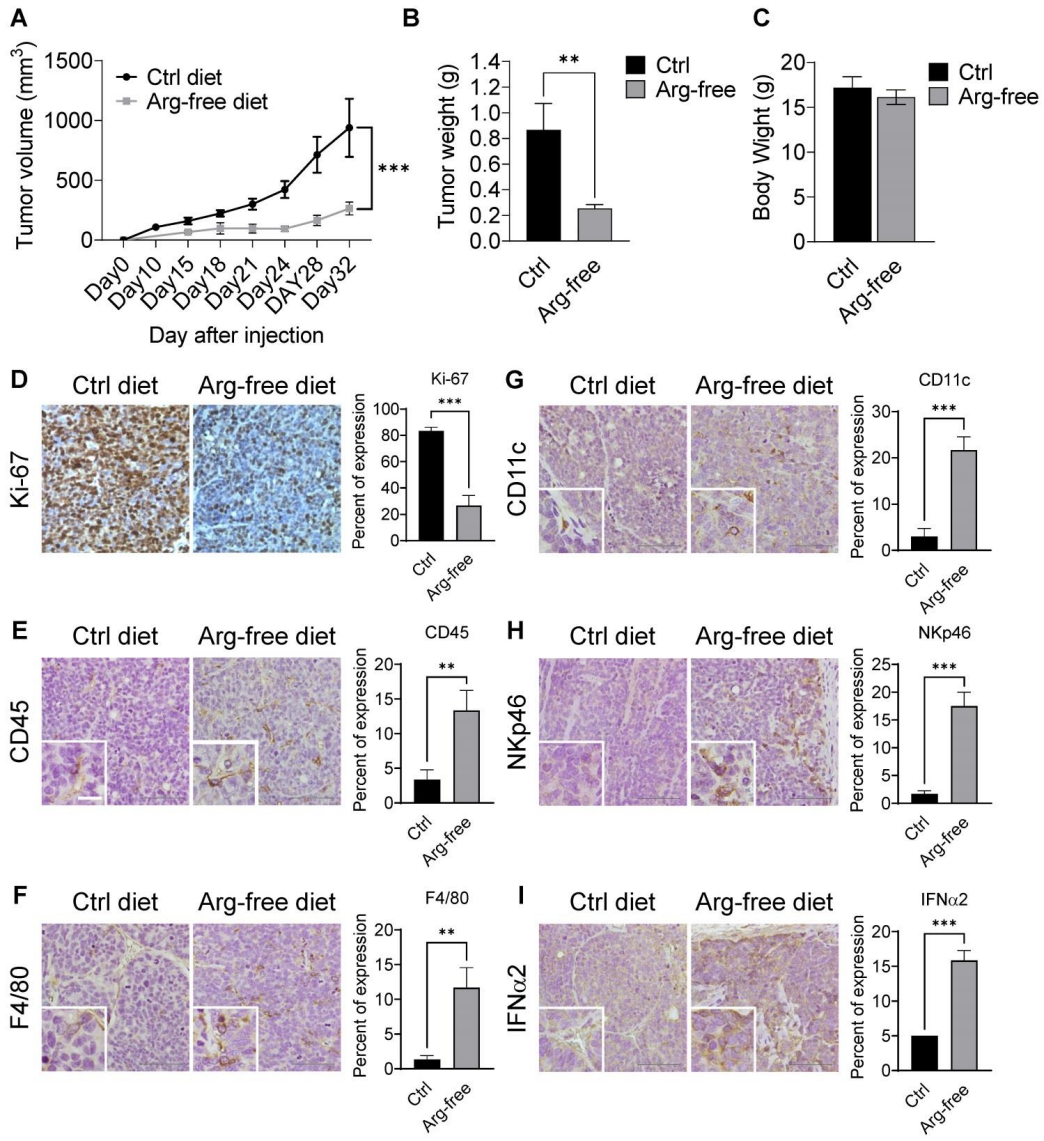
Arginine starvation induced immune cell infiltration in xenograft tumors. **(A)** Immunodeficient mice carrying CWR22Rv1 xenografts were fed either a control or arginine-free diet, and tumor growth was measured regularly. (n = 6, ***p < 0.001) **(B)** Tumor weight of xenografts. (n = 6, **p < 0.01) **(C)** Endpoint body weights of mice from both diets. (n = 3) **(D-I)** IHC staining for cell proliferation and immune cell infiltration markers. (D) Ki-67, (E) CD45, (F) F4/80, (G) CD11c, (H) NKp46, and (I) IFNα2.

CWR22Rv1 xenografts were implanted into mice fed diets either supplemented amino-acid or arginine-free. Consistently, we found that treatment with an arginine-free diet significantly reduced tumor growth as compared with the normal diet control (Figures 8A-B and S7A-B) but had no effect on body weight (Figure 8C), suggesting that dietary restriction may slow CRPC growth. Xenograft tumor tissues were used for IHC analysis. As expected, tumor tissues from animals on a normal diet displayed a higher Ki-67 index as compared to those on an arginine-free diet (Figure 8D).

Interestingly, arginine-starvation seems to induce enhanced inflammatory responses with significantly higher infiltration of innate immunity cells. Immune-markers increased in tumor tissues from animals on the arginine-free diet include anti-CD45 (a pan-leukocyte marker) (Figure 8E), F4/80 (a macrophage marker) (Figure 8F), anti-CD11c (a dendritic cell marker) (Figure 8G) and anti-NKp46 (a NK cell marker) (Figure 8H). In those tumors, secretion of IFNα2 was also markedly induced (Figure 8I). In addition, the ligands for activating NK cells, ULBP1 and ULBP3, which play an importing role in the NK-mediated immune response to tumor regression, were also upregulated in the arginine-free diet treated tumors (Figure S7C). To assess whether the activation of cGAS-STING pathway can be induced by arginine starvation* in vivo*, we measured the levels of cGAMP and dimerization of STING in the arginine-deprived xenografts. We found that both the levels of cGAMP and STING dimerization were increased in the arginine-deprived tumors (Figures S7D-E). Strikingly, we also found that the arginine-free diet-induced tumor growth suppression can be partially reversed by RU.521, with consequent decrease of arginine-deprivation-induced cGAMP levels (Figures S7F-G). The results suggested that cGAS-STING activation regulates arginine deprivation-induced tumor growth.

Taken together, our data demonstrated a causal link between arginine starvation which causes nuclear DNA leakage and the activation of DNA sensing-dependent cGAS-STING signaling which induces type I interferon production, innate immune cell infiltration and NK cell ligand expression, thereby stimulating anti-tumor immunity. Type I interferons have been shown to play a role in tumor suppression. Approved for cancer treatment 30, IFNα can enhance nature killer (NK) cell peripheral maturation in the spleen and increase its cytolytic activity toward tumors 31, 32, consistent with the observed increases in recruitment of immune cells in our xenograft experiments. Thus, our results highlight arginine restriction not only starves cancer cells but also enhances cell killing through inflammatory modulation of immune cells in the tumor microenvironment.

**Figure 9:**
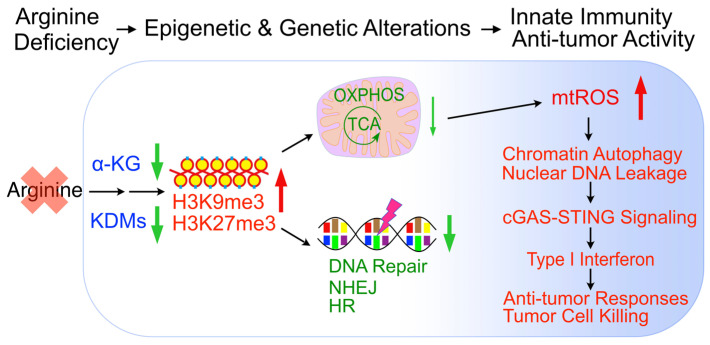
Model of arginine starvation-induced cGAS-STING activation through downregulating metabolic and DNA-repair genes and eliciting nuclear DNA leakage and chromatin autophagy.

In sum, arginine starvation induces rapid metabolite depletion affecting mitochondrial functions and histone demethylase activities, resulting in epigenetic silencing of OXPHOS and DNA-repair genes. The combined effects are the products of excessive ROS and unrepaired DNA damage, which leads to nuclear chromatin leakage catalyzed by aberrant autophagy, activation of the cGAS-STING pathway and type I interferon production (Figure 9).

## Discussion

As a semi-essential amino acid, arginine has been used as both health supplement and therapeutic target 33. Arginine-synthesis deficiency is among the most frequently observed tumor cell phenotypes and has been exploited therapeutically 29. The second-generation arginine starvation therapeutic agent ADI is under clinical trials for a variety of cancers and has an excellent safety profile 17, 21, 34. Despite its medical importance, the processes behind how arginine starvation induces tumor cell killing remain incompletely understood.

We previously showed that ADI treatment of ASS1-low prostate and pancreatic cancer cells induced atypical cell death with nuclear DNA leakage and chromatin autophagy 2. We showed that ADI induced excessive autophagy and super-sized autophagosomes, which fused with nuclear membrane to extract broken chromatin out of nucleus. This is fueled by mitochondria dysfunction and ROS production, but the mechanistic connections between these phenomena are unclear. In addition, in previous studies we have focused primarily on the cellular and therapeutic aspects of ADI treatment, which while effective at metabolizing external arginine, also generates citrulline and ammonia. The potential effects of these compounds in cell killing cannot be completely excluded. In the present work we used arginine-depleted media *in vitro* and arginine-deficient diet *in vivo* to directly examine the effects of arginine starvation, and were able to validate previously reported ADI results, thus confirming that observed phenotypes are indeed due to the removal of arginine.

We found a primary target of arginine-starvation is mitochondria, which undergo dynamic morphological changes and the depletion of mitochondrial metabolites including αKG as early as 8 to 48 h (present study and Chen CT et al. 35). This is accompanied by an impaired OXPHOS function and a coordinated silencing of the nuclear encoded OXPHOS genes. The suppressed expression of OXPHOS genes correlates with heightened levels of repressive histone markers (H3K9me3 and H3K27me3) on the promoters of these genes, instead of a decrease of mitochondria mass (Figure S8), indicating an epigenetic nature of gene silencing. We showed that depletion of αKG is critical for the epigenetic silencing, as addition of DMKG (dimethyl-α-ketoglutarate), a penetrable αKG, partially or fully restored the expression of OXPHOS genes and the low-H3K9me3 and H3K27me3 states of the promoters of these genes. DMKG also relieved the growth inhibition of the cells under arginine starvation as well as reduced the level of leaked DNA. αKG is a critical factor of Jumonji C domain-containing lysine demethylases and its depletion suppresses the activities of these demethylases, thereby increasing overall levels of histone methylation. Indeed, several histone methylation markers are increased, with H3K9me3 and H3K27me3 most prominently elevated. Similar results were reported by Pan et al. 36 during glutamine starvation. Additional report by Gabra et al. demonstrated that in melanoma, intratumoral glutamine-dependent αKG regulates the level of H3K4me3 37. The differential effects of lysine demethylases on histones could be due to their differential affinities toward αKG. In the case of H3K4me3, we did observe a modest increase but not as striking as with H3K9me3 and H3K27me3. It is possible that LSD1 and 2, histone demethylases which do not depend on αKG as cofactor may decrease the overall level of H3K4me3. TET2, a methylcytosine dioxygenase, responsible for demethylation of DNA also requires αKG, whose inhibition may also contribute to the gene silencing phenotypes. Whether this is the case awaits further analysis. Interestingly, a recent study by Morris et al. showed a high level of αKG could globally hypomethylate pancreatic cancer genome to suppress tumor growth via activating TET2 38. In our case, we found a sub-optimal level of αKG which inhibited KDMs and caused global accumulation of histone methylation, killed tumor cells. These studies underscore the importance of αKG as a key epigenetic regulator of cancer transcriptome and demonstrate that its concentration dictates cancer cell growth and survival. A consequence of H3K9me3 accumulation due to the lack of αKG is the downmodulation of DNA-repair genes. It may also mask the recruitment of DNA repair complex to the damaged site, as elegantly shown by Sulkowski et al. 39. Either case would result in impaired DNA repair with significant genome damages.

The consequence of impaired OXPHOS is the generation of ROS, which leads to DNA and nuclear membrane damage. This, coupled with slow DNA repair caused by the epigenetic suppression of nucleotide synthesis and DNA-repair genes, gives rise to chromatin leakage into cytosol, a striking feature of arginine starvation. We know little about the nature of this leaked DNA. We show that leaked DNA represents damaged chromatins associated with DNA repair enzymes and factors including DNA-PK, 53BP1, and PARP, as well as γH2AX, the phosphorylated histones deposited at DNA damage sites. It thus appears that some of the leaked DNA species are damaged nuclear DNA under repair.

What causes nuclear DNA leakage into cytosol upon arginine starvation? There are several possibilities. We previously showed that arginine depletion induced aggressive and super-sized autophagosomes, which fused to damaged nuclear membranes and extracted broken chromatins into cytoplasm, a process referred to as chromatin-autophagy or chromatophagy 2, 23. A second possibility is through micronuclei generated by genotoxic stress during mitosis. These micronuclei are enclosed by a defective nuclear membrane connected to ER 40 and decorated with γH2AX and 53BP1 41, features also detected in this study. On the other hand, arginine-deprived cancer cells usually do not go through cell division and mitosis. As such micronuclei are unlikely to account for the majority of DNA leakage after prolonged arginine-deprivation. Another route for chromatin leakage was through degradation of the nuclear membrane. It was recently reported that during Ras-induced senescence, lamin B1 was degraded through autophagy, causing damage to nuclear membrane integrity and chromatin leakage 42. Thus, a combination of excessive autophagy and genome instability is likely the root cause of chromatin DNA leakage upon arginine-depletion.

Regardless of the detailed mechanism of chromatin DNA leakage, our data showed that arginine-starvation induced nuclear DNA leakage is triggered by OXPHOS impairment. Interestingly, this is echoed by the recent work of Vizioli et al. 43 where they showed senescence-induced cytoplasmic chromatin fragments were also initiated by mitochondrial OXPHOS dysfunction. A consequence of nuclear DNA exit into cytosol is the triggering of cGAS-STING activation. We provided strong evidence that this indeed occurred during arginine starvation. Supporting evidence includes elevated expression of cGAMP 44, the presence of cGAS puncta on leaked DNA, translocation of STING 24, activation of IRF3, TBK1 and NFκB, as well as the upregulation of type I interferon production and its transactivation potential. This leads to inflammatory responses which can best be studied *in vivo*. We therefore developed a dietary arginine-restriction model to probe the consequence of arginine starvation on implanted prostate cancer xenograft and showed that an arginine-free diet indeed significantly slowed down the growth of CWR22Rv1 xenograft with little body weight loss. Importantly, the tumors in arginine-free diet-fed animals expressed elevated level of IFNα2 and with significant infiltration of immune cells as compared to those in animals fed a regular diet. This is especially significant considering that arginine is generally known as an activator of immune cells 45-47. It is, however, consistent with recent reports that ADI treatment induces immune cell infiltration in a mouse melanoma model 48. Our data thus provide new insights into the effects of arginine-starvation on the tumor microenvironment. They also suggest that arginine starvation not only starves the cancer cells but also modifies the immune environment to enhance tumor cell killing.

## Materials and Methods

### Cell culture and transfections

CWR22Rv1, PC3 and MDA-MB-231 cell lines were obtained from Bioresource Collection and Research Center, Hsinchu, Taiwan. Luciferase-overexpressing CWR22Rv1-Luc2 cells were gifted from Dr. Pei‐Wen Hsiao (Academia Sinica, Taiwan) 1. CWR22Rv1 and PC3 cells were maintained in an RPMI 1640 medium, and MDA-MB-231 in DMEM/high glucose. Both media were supplemented with 100 U/mL penicillin/streptomycin and 10% FBS. For STING-V5 and cGAS-V5 overexpressing cells, 10 μg/mL blasticidin was added. Arginine-free RMPI 1640 and DMEM/high glucose were purchased from USBiological. All arginine-free media were supplied with 10% dialyzed FBS (Gibco, 26400044), 100 U/mL penicillin/streptomycin and 10 mM HEPES. For the rescue experiment, 200 mg/L L-arginine (Sigma, A6969) was added to RPMI 1640 and 84 mg/L to DMEM/high glucose. ADI-PEG20 was obtained from Polaris Pharmaceuticals, Inc. and used at 0.2 μg/mL to deplete arginine in the media. DMKG (Sigma, 349631) was administered in 5 μM as a supplement for starvation.

### Cell viability assay

Unless otherwise noted, cell viability was determined by CellTiter-Glo (Promega, G7571). One day before starvation, cells were plated on 96-well plate at a density of 10,000-50,000 cells. For DNA leakage and Seahorse assay, media were refreshed daily to prevent nutrients depletion. Cells were lysed with CellTiter-Glo and mixed in the GloMax Discover Microplate Reader (Promega) for 2 min. Plates were incubated at room temperature for 10 min. Cell lysates were transferred to an opaque 96-well plate for luminescence measurement.

### Seahorse assay

Mitochondrial function was determined with Seahorse XFe24 Analyzer (Angilent). CWR22Rv1 cells were seeded at 5×10^4^ cells per well and cultured in arginine-deprived medium. Prior to the analysis, the culture medium was replaced with assay medium (arginine-free RPMI 1640, 2% dialyzed fetal bovine serum, 100 U/mL penicillin/streptomycin, 2 mM GlutaMAX, without sodium bicarbonate and HEPES, pH7.4) and equilibrated in a non-CO2 incubator at 37 ℃ for 1 h. OCR was measured with sequential injection of 1 μM oligomycin, 1 μM FCCP and 0.5 μM rotenone/antimycin A. Measurements were normalized to relative cell viability determined by CellTiter-Glo.

### Mitochondrial ROS imaging and quantification

Cells were stained with MitoSOX (Invitrogen, M36008) followed by a counterstain with Hoechst 33342 (Invitrogen, H3570). In brief, cells were stained with 5 μM MitoSOX for 10 min, followed by 10 μg/mL Hoechst 33342 for 15 min in culture media. After staining, live-cell images were taken using a Leica DMI6000 B microscope. To quantify MitoSOX fluorescent intensity, cells were analyzed with NC-3000 image cytometer.

### Chromatin leakage quantification

Chromatin leakage was quantified by cell counting or by ELISA assay. For cell counting, cells were fixed with 4% paraformaldehyde and stained with DAPI. Nucleus images were taken by Leica DMI6000 B microscope and counted manually. For ELISA assay, Cell Death Detection ELISAPLUS was applied in this study. Briefly, cells were lysed in 96-well plates and centrifuged to spin down nucleus. Supernatant containing cytosolic chromatin DNA were carefully collected for ELISA. First, leaked chromatin was recognized by biotinylated anti-histone antibodies and captured by streptavidin-coated wells. Then, HRP-linked anti-DNA antibodies were used to detect the captured chromatin. The color developed was proportional to the amount of leaked chromatin, and the signal was normalized to the relative cell viability.

### RNA extraction and transcriptome analysis

RNA was extracted with RNAspin Mini RNA Isolation Kit (GE Healthcare, 25-0500-72) for microarray and RT-qPCR. Gene expression difference was determined with Clariom™ S Assay, human (Affymatrix). Fold changes in gene expression were subjected to gene set enrichment analysis (GSEA). The Molecular Signatures Database (MSigDB) used in this research included hallmark and curated gene sets. The gene list for interferon-stimulated genes was based on the study by Hubel et al. 2. RNA was reverse transcribed with iScript™ Reverse Transcription Supermix (Bio-Rad, 1708841), and qPCR was carried out with GoTaq® qPCR Master Mix (Promega, A6002) on the CFX96 real-time PCR detection system (Bio-Rad). Primers are listed in Table 1.

### Western blots

Cells were lysed with RIPA buffer (25 mM Tris, pH7.4; 150 mM NaCl; 1% NP-40; 0.5% sodium deoxycholate and 0.1% SDS) supplemented with protease inhibitors (Roche, 4693132001) and phosphatase inhibitors (Roche, 4906845001) and sonicated with Bioruptor Plus at High setting. Cell lysates were resuspended in 4× sample buffer (200 mM Tris, pH 6.8; 8% SDS; 40% glycerol; 0.4% bromophenol blue and 20% 2-mercaptoethanol) and heated at 95 ℃ for 5 min. Antibodies used were OPA1 (67589), DRP1 (8570), Mitofusin-1 (14793), Mitofusin-2 (11925), H2AX (2595), γH2AX (2577), H3 (9715), H3K4me1 (5326), H3K4me3 (9751), IRF3 (11904), p-IRF3 (S396) (29047), TBK1 (3504), p-TBK1 (S172) (5483), p65 (8242) and p-p65 (S536) (3033) from Cell Signaling; H3K4me2 (39679), H3K9me1 (39887), H3K9me2 (39683), H3K9me3 (39765), H3K27me1 (61015), H3K27me2 (39919), H3K27me3 (39155), H3K36me1 (61351), H3K36me2 (39255), H3K36me3 (61101) from Active Motif; α tubulin (sc-23948) from Santa Cruz; Lamin A/C (GTX101127) from GeneTex. α tubulin was in 1:20000 dilution. For Lamin A/C and all histone-related antibodies, antibodies were used in 1:5000 dilution. Others were in 1:1000 dilution.

### Immunofluorescence

Cells were grown on cover slides and treated with arginine starvation as described. Cells were fixed with 4% paraformaldehyde for 10 min at room temperature and incubated in a blocking buffer (PBS; 5% goat serum; 0.3% Triton™ X-100) for 60 min. Antibodies used were TOM20 (1:200, 42406), γH2AX (1:400, 2577), p-53BP1 (S1778) (1:100, 2675), p65 (1:400, 8242) and V5 (1:500, 80076) from Cell Signaling; H3K9me3 (1:1000, 39765) and H3K27me3 (1:500, 39155) from Active Motif; p-DNA PKcs (S2056) (1:200, ab124918) and p-ATM (S1981) (1:100, ab81292) from abcam. Alexa 488 and Cy3 conjugated secondary antibodies (Jackson Laboratories) were used in 1:1000 dilution at room temperature for 1 h. Nuclei were stained with 0.5 μg/mL DAPI for 10 min. Immunofluorescence images were taken using a Leica TCS SP5 confocal microscope.

### ChIP-seq and ChIP-qPCR

Chromatin immunoprecipitation (ChIP) was performed with Magna ChIP™ A/G (Millipore, 17-10085). Briefly, cells were fixed with 1% formaldehyde for 10 min at room temperature followed by glycine quenching for 5 min. Chromatin was sheared to 200-1000 bp with Bioruptor Plus sonicator. Each immunoprecipitation was performed with 10 μg anti-H3K9me3 (Active Motif, 39765), anti-H3K27me3 (Active Motif, 39155) and 1 μg IRF3 (Cell Signaling, 4302) antibodies. Eluted DNA was quantified with qPCR using primers listed in Table 1. For ChIP-seq, a library was prepared using a KAPA Hyper Prep Kit and sequenced with Illumina HiSeq 2500 (50 bp, single-end sequencing). BWA was used to map trimmed reads to the human reference genome (UCSC hg38). Mapped reads were analyzed with SICER algorithm from Phalanx Biotech Group and visualized with IGV.

### Metabolite analysis

Cells were seeded at density of 4×10^6^ per 6 cm dish and subjected to arginine starvation the next day for indicated durations. After starvation, cells were washed twice with cold PBS and quenched with 1 mL -80 ℃ 80% methanol/water. Dishes were incubated at -80 ℃ for 15 min to quench metabolism. Cell lysates were scraped off and snap-frozen with liquid nitrogen followed by a vigorous vortex for complete cell lysis. Cell debris was removed by centrifugation (16,100 g for 10 min at 4 ℃), and the supernatants were collected. Supernatants were dried using SpeedVac and dissolved in ddH2O. Dissolved metabolites were centrifuged in 12,000 g for 30 min for further debris removal. The supernatant was analyzed with Waters Acquity UPLC-Xevo TQXS (Waters Corp.). Chromatographic separation was performed on a Waters ACQUITY BEH C18 column (2.1 mm × 100 mm × 1.7 mm, Waters corp.) at 45 ℃ and with elute A (water with 10 mM tributylamine and 15 mM acetic acid) and elute B (50% acetonitrile with 10 mM tributylamine and 15 mM acetic acid). The gradient profile was as follows: keep 4% B, 6 min; linear gradient 4-50% B, 0.1 min; 50%-60% B, 2.9 min; 60-100% B, 0.8 min, and keep 2.2 min. The column was then re-equilibrated for 3 min. Mix QC sample (a mixture of all samples) were prepared for analyzed during the analytical runs after every 10th sample. Mass was operated in negative mode and positive mode with multiple reaction monitoring. The parameters were as follows in both positive and negative mode: desolvation temperature was set at 500 ℃ capillary 1000 V, source temperature was set at 150 ℃; desolvation gas flow was set at 1000 L/h.

### IFNβ ELISA and luciferase assay

For INFβ luciferase assay, cells were seeded in 6-well plates and transfected overnight with pGL4.26-PRDIII-I and pRL-TK plasmids in a 50:1 ratio with TransIT-X2 (Mirus) and subjected to arginine starvation. Luciferase activities were measured using the Dual Luciferase Reporter Assay System (Promega, E1960). For IFNβ measurement, conditioned medium was collected at each time-point, and IFNβ was measured by the Human IFN-beta ELISA Kit (R&D, 41410). IFNβ concentration was normalized to total cell number.

### Immunoprecipitation

Cells were lysed with IP lysis buffer (25 mM Tris, pH7.4; 150 mM NaCl; 1% NP-40, 1 mM EDTA, 5% glycerol; protease and phosphatase inhibitors). A total of 300 μg lysates and 1 μg antibodies were used in each immunoprecipitation. IRF3 was immunoprecipitated with the IRF3 antibody (Cell Signaling, 11904) or control rabbit IgG (Millipore, pp64), and the precipitated immune complex was detected with IRF3, p-IRF3 (S396), TBK1 and p-TBK1 (S172) antibodies.

### Cytosol/nuclear fractionation

Cells were harvested by trypsinization and swelled in hypotonic buffer (20 mM HEPES, pH7.4; 10 mM KCl; 1.5 mM MgCl_2_; 1 mM DTT; protease and phosphatase inhibitors) for 10 min at 4 ℃. Swelled cells were dounced 20 times with the pestle B. Cell lysates were centrifuged at 16,000 g for 5 min at 4 ℃, and supernatants (cytosolic fraction) were collected. The pellets were washed once with the hypotonic buffer and lysed with RIPA buffer.

### Xenograft models and immunohistochemistry

Six 6-week-old immunodeficient male mice (BACL/CAnN.Cg-*Foxnl^nu^*/CrlNarl) were subcutaneously injected with 5×10^6^ luciferase-expressing CWR22Rv1 cells. Mice were divided into two groups for control (Teklad, TD.01804) and arginine-free diets (Teklad, TD.09152). Mouse diets were started 2 weeks before tumor inoculation allow for habituation. Body weight and tumor size were measured regularly. Tumor size was calculated by the equation (L×W^2^)/2 (L representing tumor length and W tumor width). Mice were sacrificed until either group reached a tumor size 20 mm in diameter. Tumor growth was evaluated by the IVIS system (IVIS Lumina II, Caliper Life Sciences) before euthanasia. All studies followed the protocol (NHRI-IACUC-106077) approved by the NHRI IACUC. Tumors were fixed with formalin for 48 h and subjected to sectioning and staining. Briefly, tumor sections were deparaffined and rehydrated. Then sections were blocked with 3% hydrogen peroxide/methanol followed by antigen retrieval with a 10 mM sodium citrate buffer at 95 ℃ for 10 min. Antibodies used were Ki-67 (Thermo, MA5-14520), CD45 (Cell Signaling, 70257), F4/80 (Cell Signaling, 70076), CD11c (Cell Signaling, 45581), IFNα2 (abcam, 193055) and NKp46 (R&D, AF2225).

### Statistical analysis

Data was presented as mean ± SD and analyzed with unpaired, two-tailed Student's t-test. Two groups were considered statistically different when p < 0.05. Unless otherwise indicated, *p < 0.05, **p < 0.01, ***p < 0.001, ****p < 0.0001. Some symbols for p values were adjusted and stated separately in figure legends.

### Data availability

Microarray and ChIP-seq data are available on the GEO website. The accession number is GSE151855.

## Supplementary Material

Supplementary figures and tables.

## Figures and Tables

**Table 1:** Primers used in the qPCR

Gene	Forward	Reverse	Purpose
NDUFA9	TTGCTTTCGTTGGTCCCAGT	CGATAGGCAAAAAGCGGCAA	RT-qPCR
NDUFB5	TGGGATTCCAGTAGCAATTTTCATA	ATTACGGGCAATCCATCTTGA	RT-qPCR
NDUFB6	AGGAAAATGGTCCATGGGGT	ACCAGGGAATATTCTGGACTTCT	RT-qPCR
NDUFC1	TTTAAGGAGCAGAGGCGGAG	GCCCATTTCATCGGTCAAGC	RT-qPCR
NDUFS3	AACCTGTTGTCTCTGCGCTT	CAGTTGGCTGCCTTGAACAC	RT-qPCR
NDUFS6	GTTCAGACAGCACCACCACT	CACCAGGAATACCCTTCGCA	RT-qPCR
NDUFS7	GCAGCTGCAGAGGAAGATCAA	CAACCTCACGGGACACAAGC	RT-qPCR
SDHA	AAACTCGCTCTTGGACCTGG	TCTTCCCCAGCGTTTGGTTT	RT-qPCR
UQCRFS1	ACCCAGTTCGTTTCCAGCAT	CAGGGGTTTGCCTCTCCATT	RT-qPCR
COX5A	TGGCTATCCAGTCAGTTCGC	CCTTTACGCAATTCCCAGGC	RT-qPCR
COX18	AGCCACTGTGTTGGTTTGGA	CTGCTTCTGAATGTGCTGCC	RT-qPCR
ATP5D	AGCCAAGGCAAACTTGGAGA	CTGGCTCGCCTACTCCAG	RT-qPCR
ATP5G1	GGCTAAAGCTGGGAGACTGA	AAGAAGGAGGCAGACACAGG	RT-qPCR
ATP5J2	AATGGCGTCAGTTGGTGAGT	GGACTGAAGTCCCGCATCAA	RT-qPCR
AK5	AGAGTTCGGACGCAGGATTG	ATGGTCTTGGTGGTGTCGTC	RT-qPCR
AK7	GACTTTGCGGTGGAGACGTA	CACTGAGTGCAGAGACTGCC	RT-qPCR
DHODH	GGAGACACCTGAAAAAGCGG	CAGAGTCGGCATCAGGTGTT	RT-qPCR
RRM1	CTTCCCTGGCCCTGAATATGT	GGCATGCCTCTGGTACAGG	RT-qPCR
RRM2	TGGTCGACAAGGAGAACACG	TTAGTTTTCGGCTCCGTGGG	RT-qPCR
FEN1	CCAAAGGCCAGTCATCCCTC	GCATCAATGGCCACCTTACG	RT-qPCR
DCLRE1C	TCAGATTGGCGCAAGGAGAA	ACACTCCTCCCGACTTGGAA	RT-qPCR
NBN	GGCCATCCCAGTACAGGATTA	GGGCGCTTGGCATTAGTTTT	RT-qPCR
FANCD2	TCCCAAAATTCCCAGGAGAGC	AATGCAACCATCAGTGCCAGA	RT-qPCR
FANCG	AGCCTCACCCCTTCATTGTG	CTTGGCTCCGAGCTATCCAG	RT-qPCR
FNACI	ACCCTGAGAGAAGGTGATTTGAC	GAGCAGGGGGAACCTTTGAA	RT-qPCR
FAAP24	GGATGGCTTGACACCAGACT	CTGCCACCAAATCAGCTTCG	RT-qPCR
BRCA1	GTATTCTGAGAGGCTGCTGCT	CGCGCAGTCGCAGTTTTAAT	RT-qPCR
BRCA2	ACAAAGGCAACGCGTCTTTC	TGAGAACACGCAGAGGGAAC	RT-qPCR
RAD51D	AGGAACAGATGGACTTTCGCT	CCTGAGTTTTGCCGCTACCT	RT-qPCR
IFNA1	ACCTAGAGCCCAAGGTTCA	GAGAGCAGCTTGACTTGCAG	RT-qPCR
IFNA2	TCGTATGCCAGCTCACCTTT	GGGACTAGTGCCTTAAGAGCTG	RT-qPCR
IFNA4	AAACCTAGAGGCCGAAGTTCAA	CAGCCCAGAGAACAGATGGA	RT-qPCR
IFNA6	ACCTTCAACCTCTTCAGCACA	TCAGTATAGAGTTTGTCTAGAAGCC	RT-qPCR
IFNA7	CACCTCAGGTAGCCTAGTGAT	CTATTACGCAGGCTGTGGGT	RT-qPCR
IFNA8	AGCAGCTCACACTTCGACAA	GGGACTAGTGCCCATACAGC	RT-qPCR
IFNA10	TGAAGGACAGACATGATTTCCGAAT	GCTTGAGCCTTCTGGAACTG	RT-qPCR
IFNA14	GTTACCCCTCATCAACCAGCC	CAGAGAGCAGCTTGACTTGC	RT-qPCR
IFNA16	GCTCAAGCCATCTCTGCCTTC	TTCTAGGTCATTCAGTTGCTGGAA	RT-qPCR
IFNA21	GGGAGGTTGTCAGAGCAGAAA	AGTCATAGAAGTGTGGACTGGTG	RT-qPCR
IFNB1	TCTCCTGTTGTGCTTCTCCAC	GCCTCCCATTCAATTGCCAC	RT-qPCR
18S	AAACGGCTACCACATCCAAG	CCTCCAATGGATCCTCGTTA	RT-qPCR
NDUFB5	TTCCTCACTCGTGGCTTTCC	TGGAAGCGTCCTCCCTTTTC	ChIP-qPCR
NDUFB6	AGTGGTGTTGATCATGGTATTGT	ACATGTCTCCCCATACCAAAAA	ChIP-qPCR
NDUFA9	TCCATGGAGCAAAAAGAAATTCCA	GCCGCTTTTGAATCTGTTGC	ChIP-qPCR
NDUFS3	GTTCTGTTGCTGCCGGTGA	AGGGCAAGGACAGGCATCTA	ChIP-qPCR
SDHA	GAGGAGTCGTGTCTGCCCAA	TCAGGACAACCCGCACAGAG	ChIP-qPCR
UQCRFS1	ACCTGCAAGCAACAAAACCC	CTTCGCCTGGTTCCTCCTC	ChIP-qPCR
COX5A	TCAGTAACCAGGGTAGGGCA	CTCGTGTCCTTGCGGTGAC	ChIP-qPCR
ATP5G1	TGGGAGAGGCTATGGCAATC	CCTGCCCATCTTACACAGCA	ChIP-qPCR
ATP5J2	GAAGCGGCTATTTCTTAGCGG	TGGAGGGTGAAGGCATCTGG	ChIP-qPCR
ATP5D	CAGAGCCGGAAACTTGATGT	GATTTTTGGGGCGCTCATTG	ChIP-qPCR
IFNA14	ACTAGCTTCCATTTTCTGAACGTC	TGAGAAACTGCTCTACACCCAT	ChIP-qPCR
IFNB1	GCTCTGGCACAACAGGTAGTA	TGTAGTGGAGAAGCACAACAGG	ChIP-qPCR

Abbreviations ADI-PEG20: pegylated arginine deiminase; ASS1: argininosuccinate synthetase 1; ChIP: chromatin immunoprecipitation; cGAS: cyclic GMP-AMP synthase; CRPC: castrate-resistant prostate cancers; GSEA: gene set enrichment analysis; IFNs: interferons; KDMs: lysine demethylases; OPA1: optic atrophy-1; OXPHOS: oxidative phosphorylation; STING: stimulator of interferon response cGAMP interactor 1
