# Supplementary material for: Arginine starvation elicits chromatin leakage and cGAS-STING activation via epigenetic silencing of metabolic and DNA-repair genes
Source: Theranostics. 2021 Jun 4;11(15):7527–45. doi: 10.7150/thno.54695 (PMC8210599; doi:10.7150/thno.54695)
Supplement: Supplementary file 1 — Supplementary figures and tables. [file thnov11p7527s1.pdf]

Figure S1

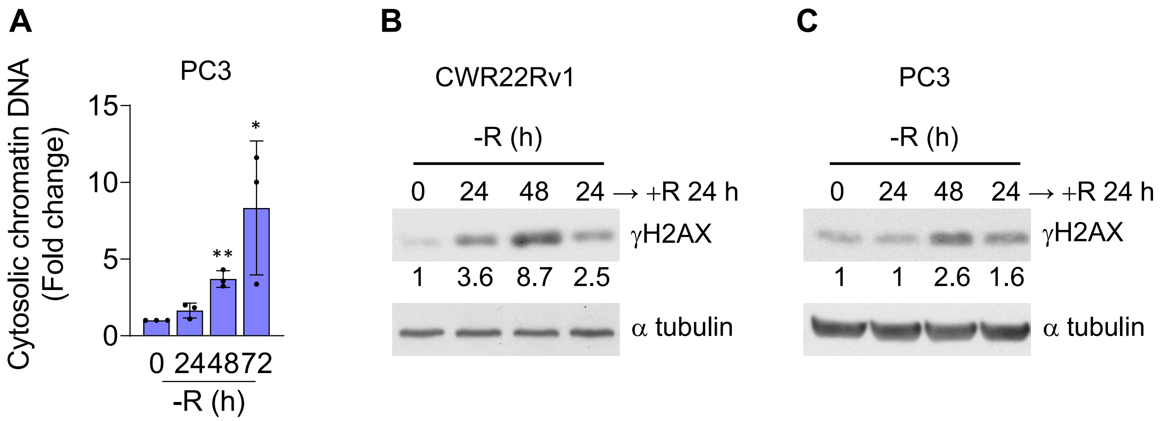

Figure S1. Arginine starvation induced DNA damage and chromatin leakage in prostate cancer cells.

(A) PC3 cells were starved for indicated timepoints, and cytosolic chromatin DNA was measured with ELISA assay. ($n = 3$, $*p < 0.05$, $**p < 0.01$)

(B) Immunoblot analysis of γH2AX in CWR22Rv1 cells deprived of arginine. Fold change is listed below the blot.

(C) Immunoblot analysis of γH2AX in PC3 cells deprived of arginine. Fold change is listed below the blot.

Figure S2

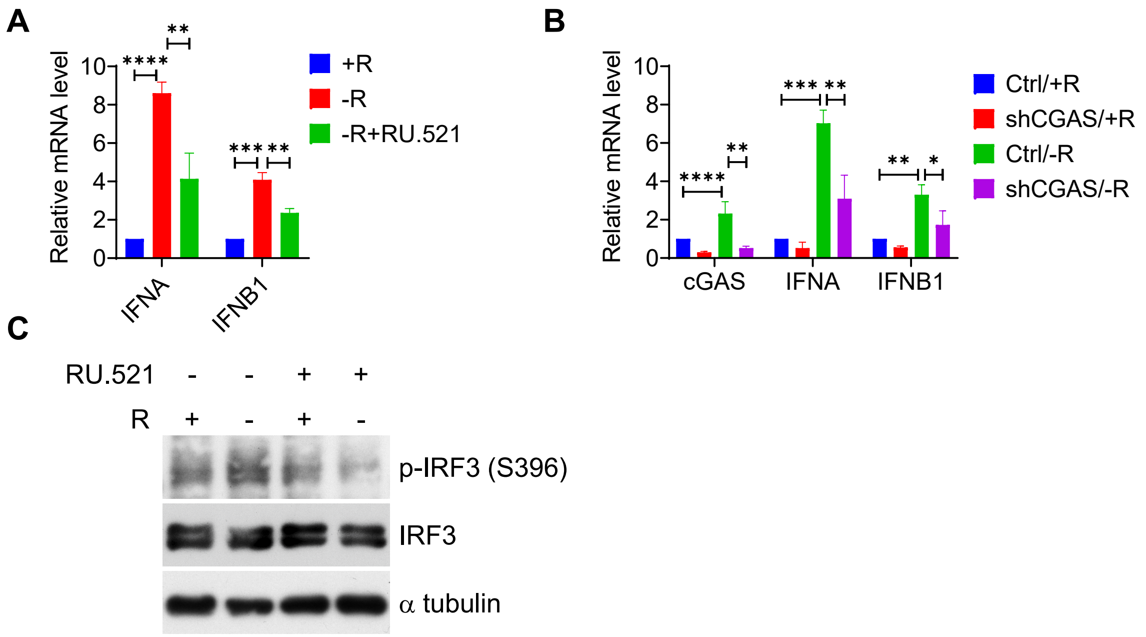

Figure S2. Inhibition of cGAS reduced the type I IFNs response.

(A) CWR22Rv1 cells were deprived of arginine with or without RU.521, and type I IFNs expression were analyzed by RT-qPCR. (n = 3, ** $p < 0.01$, *** $p < 0.001$, **** $p < 0.0001$)

(B) CGAS was knockdown by shRNA, and cGAS and type I IFNs expression were analyzed by RT-qPCR. (n = 3, ** $p < 0.01$, *** $p < 0.001$, **** $p < 0.0001$)

(C) IRF3 phosphorylation was analyzed by western blots.

Figure S3

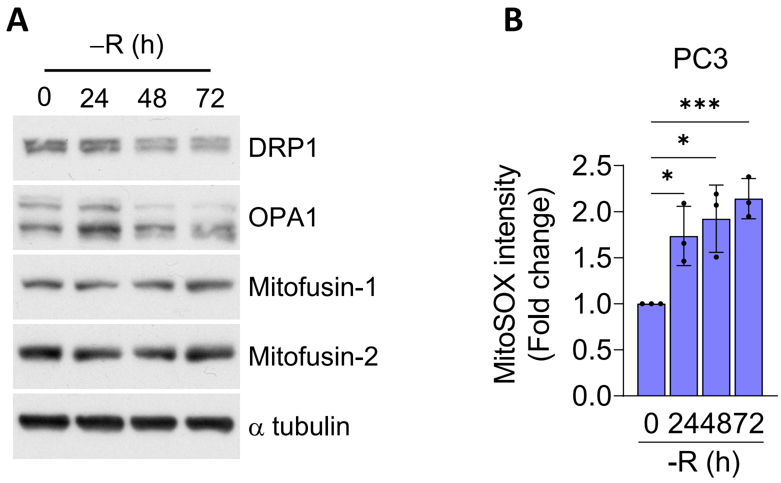

Figure S3. Arginine starvation altered mitochondrial dynamics and function.

(A) Analysis of mitochondrial dynamics regulating proteins in CWR22Rv1 cells.

(B) Mitochondrial ROS level in PC3 cell after arginine starvation. (n = 3, *p < 0.05, ***p < 0.001)

Figure S4

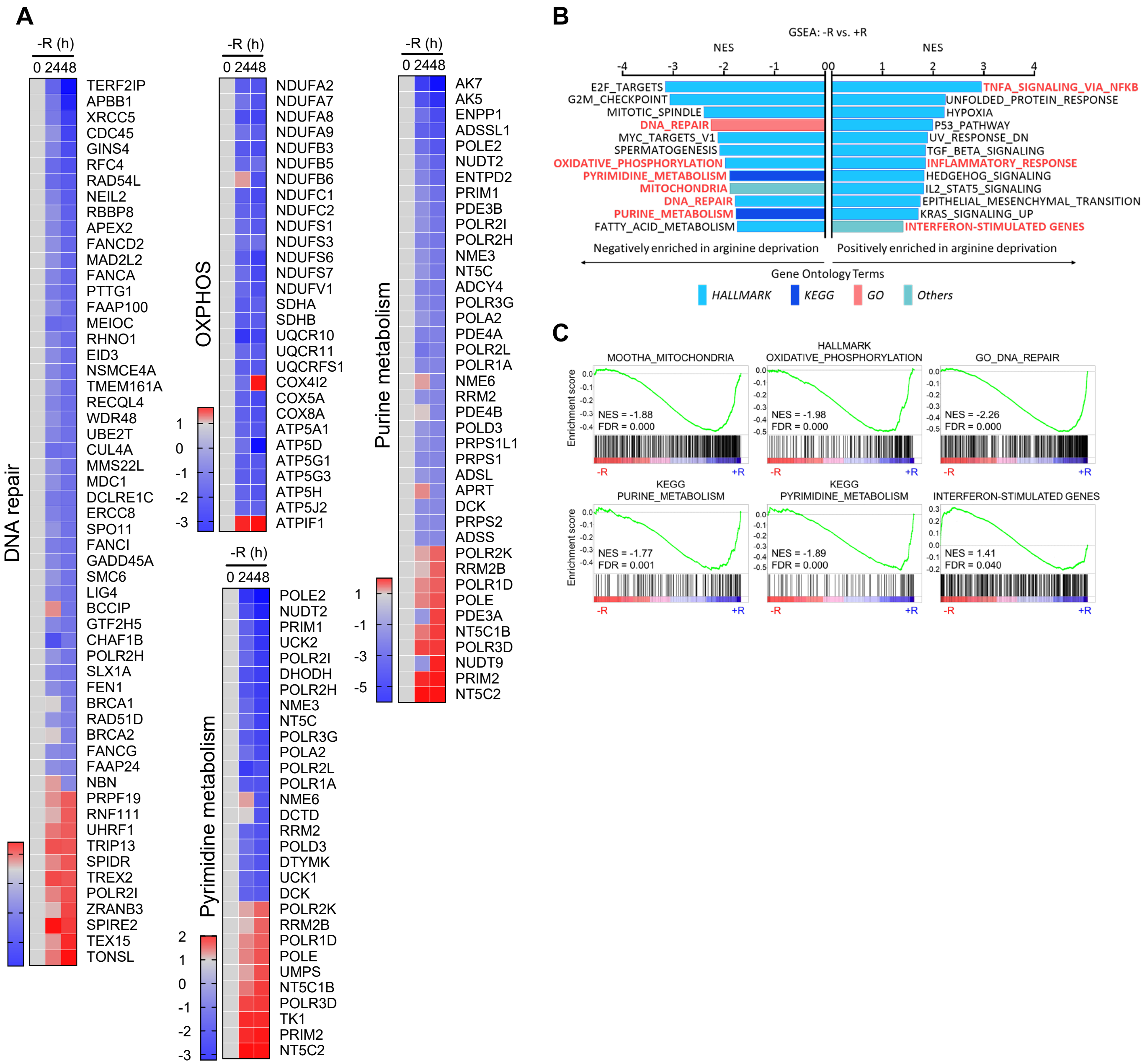

Figure S4. Arginine starvation downregulated OXPHOS and DNA-repair genes.

(A) Gene expression of metabolic and DNA repair related genes was analyzed by microarray.

(B) PC3 cells were deprived of arginine for 48 h and subjected to GSEA analysis. All enrichment results met the statistical criteria of $p < 0.05$ and FDR < 25%.

(C) Enrichment score plots of selected gene sets in PC3 cells.

Figure S5

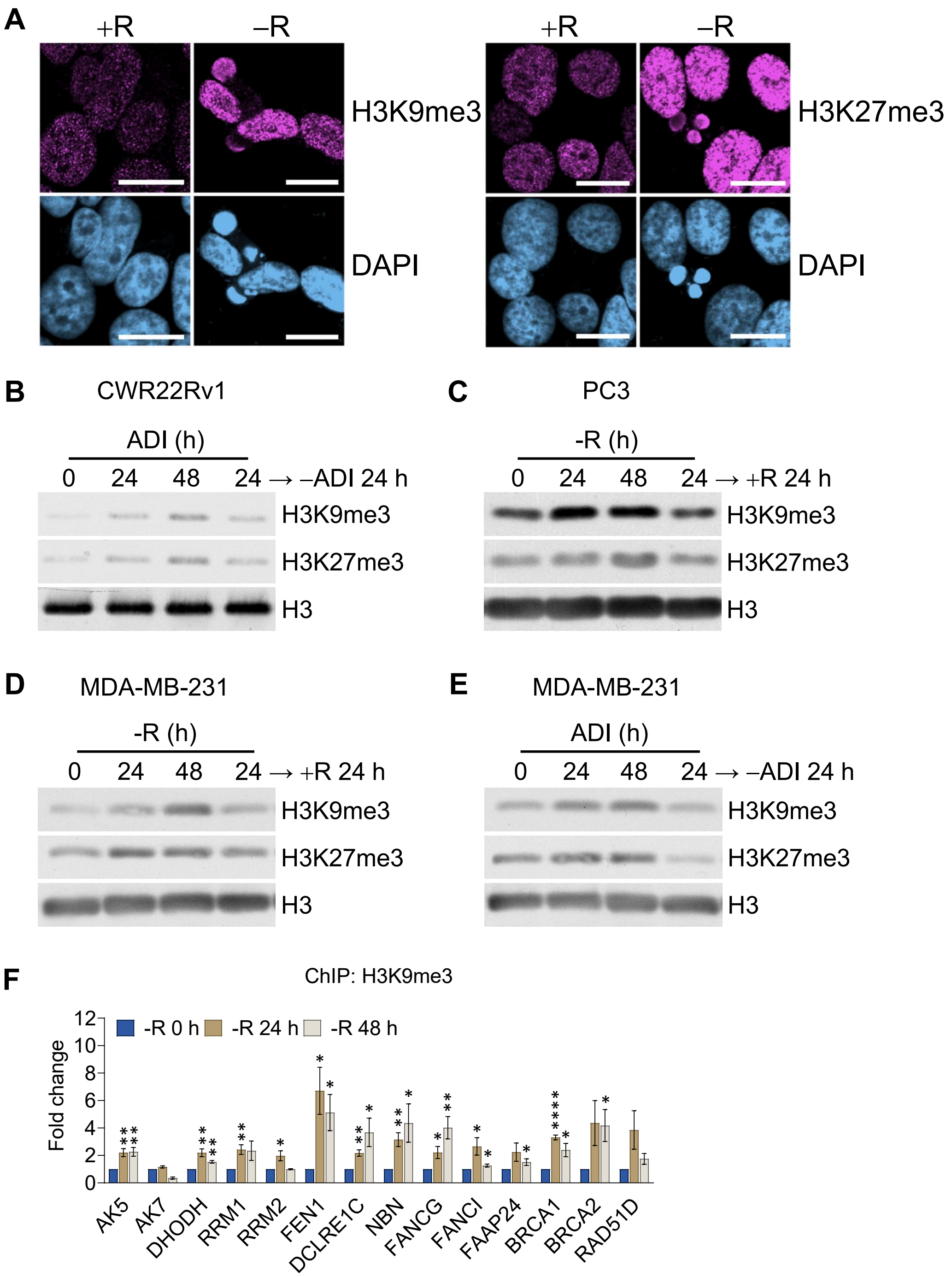

Figure S5. H3K9me3 and H3K27me3 were upregulated by arginine starvation in ASS1-low cells.

(A) H3K9me3 and H3K27me3 expressions in CWR22Rv1 cells were analyzed with immunostaining. Scale bars, 10 µm.

(B) Immunoblot analysis of H3K9me3 and H3K27me3 in CWR22Rv1 cells treated with ADI-PEG20.

(C) H3K9me3 and H3K27me3 expression in arginine-deprived PC3.

(D) H3K9me3 and H3K27me3 expression in arginine-deprived MDA-MB-231.

(E) H3K9me3 and H3K27me3 expression in ADI-PEG20-treated MDA-MB-231.

(F) H3K9me3 recruitment to the promoter regions of DNA-repair genes was analyzed by ChIP-qPCR. ($n = 3$, *$p < 0.05$, **$p < 0.01$, ****$p < 0.0001$)

Figure S6

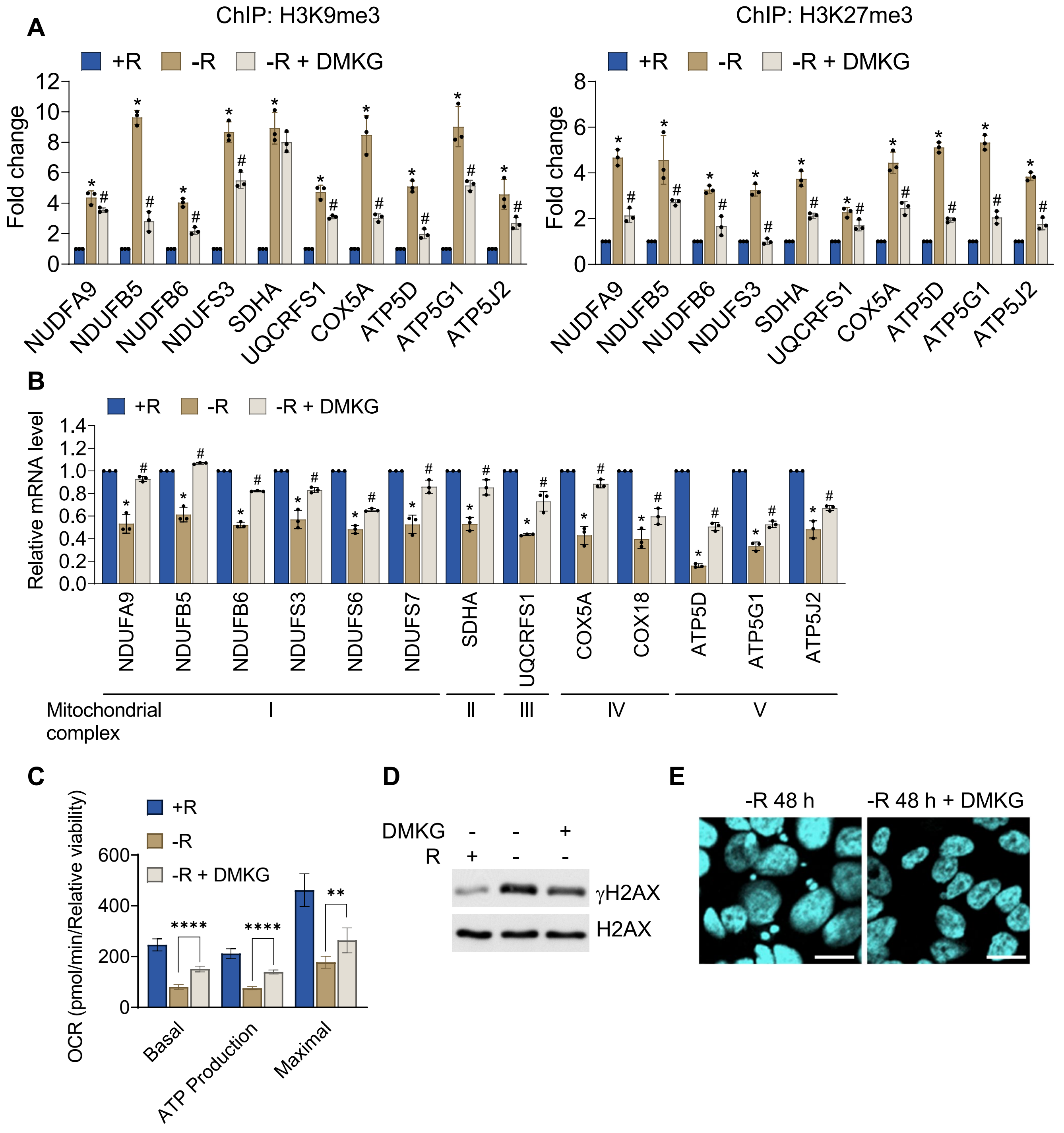

Figure S6. Supplement of αKG partially restored OXPHOS gene expression and alleviated DNA damage.

(A) H3K9me3 and H3K27me3 distribution on the promoter regions of OXPHOS genes in arginine-deprived CWR22Rv1 with or without DMKG. (n = 3, * represents -R vs +R, and # represents -R + DMKG vs -R. n = 3, $^{*,\#}p < 0.05$)

(B) OXPHOS gene expression in arginine-deprived CWR22Rv1 with or without DMKG. (* represents -R vs +R, and # represents -R + DMKG vs -R. n = 3, $^{*,\#}p < 0.05$)

(C) Basal respiration, ATP production and maximal respiration in arginine-deprived CWR22Rv1 with or without DMKG. (n = 5, **p < 0.01, ****p < 0.0001)

(D) DMKG reduced arginine starvation-induced γH2AX in CWR22Rv1.

(E) Representative images of chromatin leakage in the presence of DMKG. Scale bars, 10 μm.

Figure S7

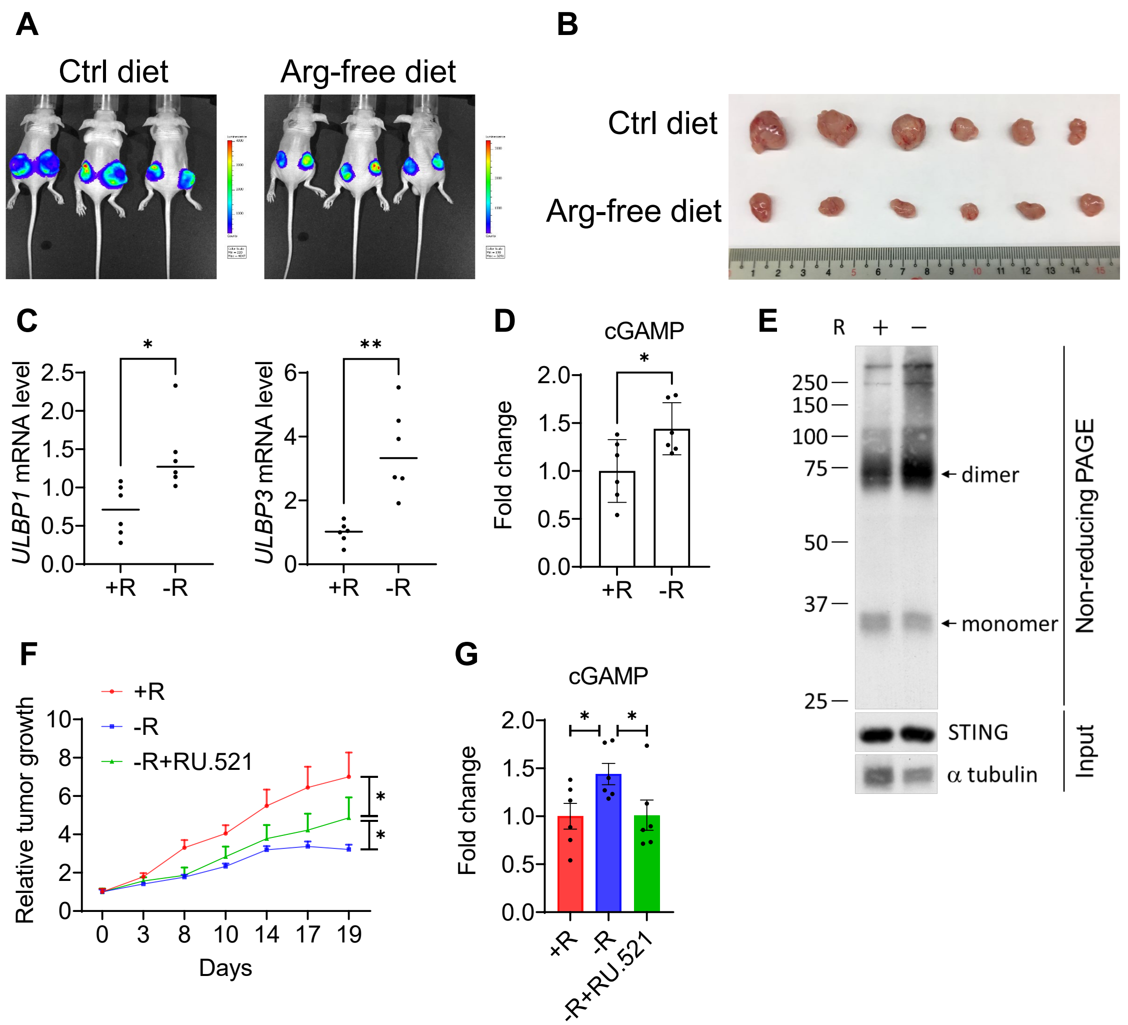

Figure S7. Arginine starvation regulated expression of NK cell ligands.

(A) IVIS images of CWR22Rv1 xenografts.

(B) Tumor images of isolated xenografts.

(C) Expression of NK cell ligands, ULBP1 and ULBP3 were analyzed with RT-qPCR. ($n = 6$, *$p < 0.05$, **$p < 0.01$)

(D) CWR22Rv1 xenografts were lysed with IP lysis buffer, and cGAMP level was determined by ELISA assay. ($n = 6$, *$p < 0.05$)

(E) Cell lysates from the xenografts were separated in non-reducing SDS-PAGE for detecting dimerized STING.

(F) Treatment of RU.521 partially rescued the tumor growth inhibition in an arginine-free diet mouse xenograft model.

(G) ELISA assay for measurement of cGAMP levels in the tumors of (F).

Figure S8

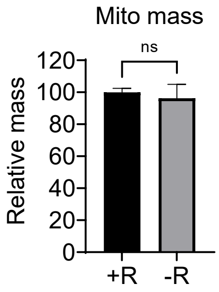

Figure S8. CWR22Rv1 cells were cultured in arginine-free medium for 72 h, and mitochondrial mass was determined using Mitotracker green. (n = 3)

Table S1

Table S1. Metabolite analysis in PC3 treated with ADI-PEG20

PC3/Metabolite	R(-)/R(+)
Quinolinic acid	2.123
L-Pyroglutamic acid	1.522
D-Glucose 6-P	1.015
D-Fructose 6-P	1.015
Tryptophan	0.993
Citrulline	0.942
Ribose 5-P	0.935
Glyceraldehyde-3-P	0.92
Phosphoenolpyruvate (PEP)	0.917
Glucose	0.912
L-Carnitine	0.899
Fructose-1,6-BP	0.859
3-Phosphoglycerate	0.853
NAD+	0.602
Glutamate	0.502
**α-Ketoglutarate**	0.402
2,3-dinor Thromboxane B2	0.18
Thromboxane B2	<0.1
Pyruvate	<0.1
Malate	<0.1
Succinate	<0.1
Lactate	<0.1
Prostaglandin D2	<0.1
11-dehydro-2,3-dinor Thromboxane B2	<0.1
